# Comparison of Streptococcus mutans growth-phase-dependent transcriptome changes in two different simulated microgravity models

**DOI:** 10.1099/mic.0.001776

**Published:** 2026-10-05

**Authors:** Ke Aira T. Davis, Kelly C. Rice

**Affiliations:** 1 Department of Microbiology and Cell Science, IFAS, University of Florida, Gainesville, Florida, 32611, USA

**Keywords:** cell clumping, physiology, RNA-Seq, simulated microgravity, *Streptococcus mutans*

## Abstract

*Streptococcus mutans* is a cariogenic member of human dental plaque biofilm. Our previous study of *S. mutans* early stationary-phase rotary cell culture system (RCCS) cultures revealed significant transcriptome changes under low-shear modelled microgravity (LSMMG) compared to normal gravity (NG), as well as an altered cell clumping phenotype. In this current study, *S. mutans* mid-exponential phase and late-exponential phase cultures were compared using the RCCS (LSMMG and NG orientations) as well as simulated microgravity (‘0 g’) using a random positioning machine (0 g RPM). The previously described stationary-phase LSMMG-altered cell clumping phenotype was also observed at earlier growth phases in LSMMG and 0 g RPM. Significant fold-change differences in gene expression (late-exponential/mid-exponential phase) were observed in LSMMG, NG and 0 g RPM, with 24 genes shared between LSMMG and 0 g RPM cultures (including upregulated pyrimidine biosynthesis genes and decreased genes involved in purine biosynthesis, iron transport and malolactic fermentation). Additional significant functional enrichments included TCA cycle (LSMMG and NG), starch and sucrose metabolism (LSMMG and 0 g RPM) and folate biosynthesis (LSMMG, 0 g RPM and NG). Overall, these data provide insight into *S. mutans* physiology in response to simulated microgravity. Further investigation of transcriptome changes shared between LSMMG and 0 g RPM may allow us to better predict how this bacterium responds to spaceflight conditions.

## Data Availability

FASTQ files and metadata are accessible through NASA’s Open Science Data Repository (https://osdr.nasa.gov/bio/repo/data/studies/OSD-987).

## Introduction

Streptococci, which make up 20% of oral bacteria, can contribute to both the early development of dental plaque and advanced tooth disease [1, 2]. *Streptococcus mutans* is a prevalent member of human dental plaque biofilm and is considered a significant pathogen in the development of dental caries, due in part to its ability to rapidly ferment a wide variety of carbohydrate sources, its ability to adapt to and survive the ensuing acidic environment and its strong biofilm-forming ability (reviewed in [3]). Although cariogenic streptococci have been extensively studied with respect to their physiology and treatment methods on Earth, these observations may not be applicable during extended space travel. For example, astronauts undergo physiological changes during spaceflight that could predispose them to caries, such as bone loss, muscle atrophy and decreased salivary lysozyme [4, 5]. In the Skylab space station studies conducted in the 1970s, astronauts displayed increased abundance of oral streptococci (*Streptococcus sanguinis*, *S. mutans*), as well as decreased salivary lysozyme production, increased dental calculus and increased gingival inflammation [5]. In a more recent spaceflight study, International Space Station (ISS) astronauts were monitored for changes in the salivary microbiome before, during and after time in space [6]. These experiments found that the overall bacterial diversity in saliva was decreased in spaceflight samples compared to pre- and post-flight samples, with the *Streptococcus* genus being most abundant [6].

In general, spaceflight experiments are difficult to conduct because of their cost, hardware requirements and time commitment from astronauts aboard the ISS. Therefore, our understanding of the physiological response of *S. mutans* and other oral streptococci to spaceflight variables such as microgravity has been restricted thus far to ground-based simulated microgravity models. As a first step, we previously compared the growth properties, stress resistance and transcriptome changes of early stationary-phase *S. mutans* low-shear modelled microgravity (LSMMG) and normal gravity (NG) cultures using the ground-based rotary cell culture system (RCCS). In this 2D clinostat model, high-aspect rotating vessels (HARVs) are rotated on a perpendicular axis to the gravitational vector to model microgravity (LSMMG), whereas NG HARVs are rotated on a parallel axis (Fig. 1a). These experiments demonstrated that LSMMG cultures grown in sucrose-containing biofilm medium (BM) exhibited distinct physiological changes, including altered cell clumping and increased sensitivity to oxidative stress, as well as significant changes in global transcriptome (as assessed by RNA-Seq) compared to NG cultures [7]. More recently, we developed a random positioning machine (RPM)-based method for growing streptococcal biofilms under simulated microgravity, which revealed that *S. mutans* simulated microgravity biofilms underwent structural changes (decreased substratum coverage, increased formation of tower-like structures) relative to NG control biofilms [8]. The RPM (Fig. 1b) is a 3D clinostat that can be programmed to simulate microgravity and other defined partial gravities. Samples are rotated on a two-axis platform with continuous changes in orientation at a speed faster than the object’s response to Earth’s gravity, thereby ‘distributing the Earth’s gravity vector in all directions over time’ [9].

**Fig. 1.**
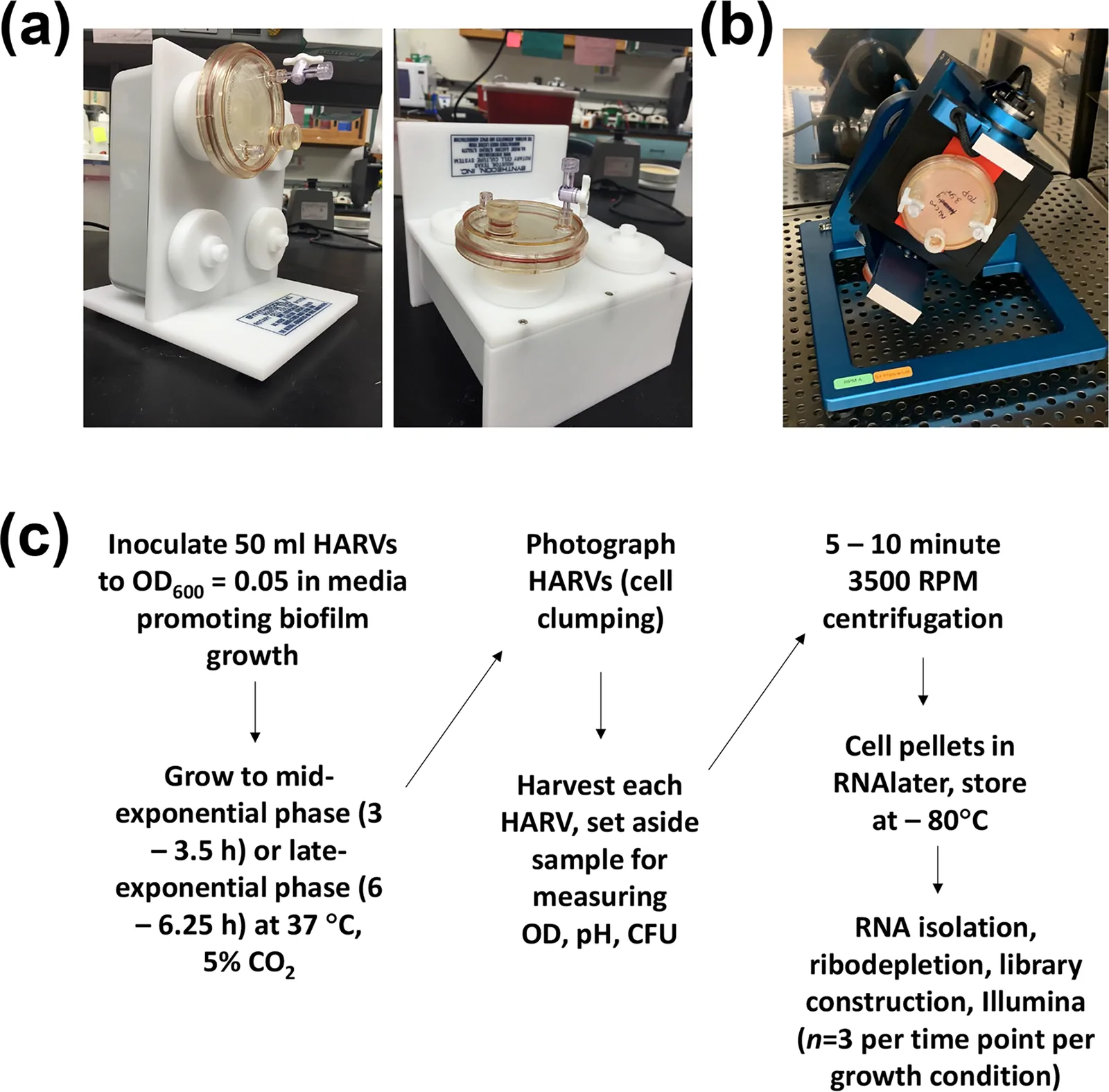
Experimental hardware and overview. (a) Photograph of RCCS NG (right) and LSMMG (left) hardware. (b) Photograph of RPM hardware. (c) Experimental workflow for both RCCS and RPM model experiments.

An important consideration in the use of ground-based models to study biological responses to simulated microgravity is a lack of concordance that can exist between changes in spaceflight-cultured microbes and changes observed in ground-based LSMMG, recently illustrated by comparing transcriptome data of several bacteria grown in both spaceflight and LSMMG conditions [10–12]. Additionally, these studies suggested that different bacterial species are not likely to share a common biological response to spaceflight and/or simulated microgravity [10–12]. Therefore, the goal of this current study was to compare *S. mutans* growth between two distinct ground-based microgravity models (LSMMG and 0 g RPM) as well as the typical RCCS NG control to determine if there are any shared phenotypes or transcriptome responses. It is anticipated that these data will provide a hypothesis-generating framework to guide the design of future spaceflight experiments with oral streptococci.

## Methods

### Bacterial strains and culture conditions

Experiments were conducted using *S. mutans* serotype c strain UA159, provided by Dr Robert Burne (University of Florida Department of Oral Biology, Gainesville, FL). All *S. mutans* cultures, including those grown in HARVs, were cultivated at 37 °C in a 5% CO_2_ incubator. For each experiment, *S. mutans* was freshly inoculated from a 40% (v/v) glycerol stock maintained at −80 °C onto brain heart infusion (BHI) agar and allowed to grow for 48 h. Prior to the inoculation of HARVs, a single colony was inoculated into 30 ml of BHI broth (Becton Dickinson BBL) in a 50 ml Falcon tube (cap slightly loose to promote gas exchange) and grown as a static culture at 37 °C and 5% CO_2_ for 16 h. For the experiments described below, RCCS experiments were conducted at the University of Florida (Gainesville, FL, USA), and RPM experiments were conducted at the Microgravity Simulation Support Facility (MSSF), NASA Kennedy Space Center (Merritt Island, FL, USA) [13].

### Inoculation and growth of HARVs

One litre of fresh biofilm medium (BM) containing 11 mM glucose and 10 mM sucrose, a semi-defined medium that promotes sucrose-dependent biofilm formation [14], was prepared for each experiment. All HARV manipulations were performed under a Class II, Type A2 biosafety cabinet to maintain sterility. Disposable 50 ml HARVs (Synthecon, Houston, TX, USA) were used for the NG, LSMMG and RPM growth models. Similar to the RCCS system, the RPM platform on which the HARVs were attached was designed to allow air exchange through the HARV oxygen-permeable membrane. For each experiment, sterile BM (~45 ml) was added to each disposable 50 ml HARV to pre-wet the oxygen-permeable membrane. These HARVs were incubated for 24 h at room temperature prior to use in each experiment. The next morning, the OD_600_ of a 30 ml overnight *S. mutans* BHI culture (grown as described above) was measured using a spectrophotometer and used to calculate how much volume of overnight culture to add to 250 ml of BM to achieve a starting OD_600_ of 0.05. Then, ~54 ml of this inoculum was used to fill each HARV, after removal of the sterile BM. The inoculum was mixed vigorously by swirling and vortexing before filling each HARV. Prior to growth on the RCCS or RPM, each inoculated HARV was inspected for air bubbles, which were removed by syringe displacement with sterile medium as described by the RCCS manufacturer (Synthecon) and in [7, 15]. Each of the 50 ml HARVs was labelled as follows: RCCS NG (mid-exponential and late-exponential phases), RCCS LSMMG (mid-exponential and late-exponential phases) and 0 g RPM (mid-exponential and late-exponential phases).

For RCCS growth, two HARVs were attached to the horizontal rotator base (Synthecon) for the NG control, and two HARVs were attached to the vertical rotator base (Synthecon) for LSMMG. Rotator bases were placed in a 5% CO_2_ incubator set to 37 °C and connected to their respective power supplies (located outside the incubator). Rotation speed was set at 30 RPM for NG and LSMMG growth, a speed previously determined to be the minimum required for counteracting sedimentation of *S. mutans* cell aggregates in the LSMMG orientation [7]. Then, HARV cultures were grown for either 3-h (mid-exponential) or 6-h (late-exponential) growth phase. The leftover inoculum was assessed for OD_600_ on a spectrophotometer, serial dilution for c.f.u. enumeration and pH using ColorpHast strips (EMD Millipore).

For RPM growth, each HARV was screwed to the centre of a base attached to the two independently rotating frames of each RPM instrument (Airbus, Leiden, The Netherlands). Each RPM was housed in a 5% CO_2_ incubator set to 37 °C and was run using ‘partial g motion mode’ set for a 0-g path to simulate microgravity. These 0 g RPM HARV cultures were grown for either 3.5 h (mid-exponential growth phase) or 6.25 h (late-exponential growth phase). The leftover inoculum was assessed for OD_600_ on a spectrophotometer, serial dilution for c.f.u. enumeration and pH using ColorpHast strips (EMD Millipore).

### Harvest and sample processing of RCCS and RPM HARV cultures

The overall experimental design for both RCCS and 0 g RPM models is summarized in Fig. 1(c). HARVs (*n*=3 independent experiments per time point per growth condition) were harvested at mid-exponential and late-exponential growth for both RCCS (LSMMG and NG controls) and 0 g RPM cultures. The time points corresponding to these phases of growth for both RCCS and RPM models were determined through previously published [7] and preliminary growth experiments (data not shown). At each harvest time point, HARVs were removed from the incubator and photographed to document cell clumping. Each HARV was then shaken vigorously to remove any adherent cell clumps from the membrane, and ~25 ml was removed and transferred to a 50 ml Falcon tube. The HARV was then closed and shaken vigorously for 30 s (to remove any adherent cells from walls of the HARV), and the remaining volume (~25 ml) was added to the initially removed volume. Each Falcon tube was then vortexed at high speed for 30 s, and 1 ml was removed and set aside to determine the OD_600_, c.f.u. ml^−1^ and pH as described above. Triplicate 15 ml aliquots of each HARV culture were then centrifuged at 4 °C for 5 min, and 1 ml RNAlater (Invitrogen-Thermo Fisher, Waltham, MA, USA) was added to cell pellets after decanting the culture supernatants.

### RNA isolation and sequencing

Total RNA was isolated from *S. mutans* frozen cell pellets using the Qiagen RNeasy kit followed by treatment of isolated RNA with Turbo-DNase (Invitrogen-Thermo Fisher), as described in [7]. Each RNA sample was subjected to TapeStation analysis to determine the RNA integrity number (RIN) to ensure RNA samples had sufficient integrity to proceed with ribodepletion, library construction and sequencing. The RIN values for all of the RCCS and RPM samples in this experiment (both ME and LE phase samples) ranged from 8.9 to 9.6. Samples were then submitted to the University of Florida Interdisciplinary Center for Biotechnology Research (ICBR) Gene Expression and Genomics core facilities for ribodepletion, cDNA library construction/barcoding and sequencing. The Illumina Stranded Total RNA Prep with Ribo-Zero Plus kit was used for each RNA sample. Barcoded sample libraries were sequenced on an Illumina NovaSeq6000, S4, 2×150, one lane (750 Gb). FASTQ files (36–59 million reads per sample) were generated from each set (18 samples total) of paired-end reads, and data was delivered through Illumina Base Space.

### RNA-Seq and differential expression analysis

FASTQ sequences for each RNA-Seq sample were downloaded from Illumina BaseSpace and imported as paired-end reads into CLC Genomics Workbench version 25 (Qiagen Digital Insights, Redwood City, CA, USA), which was used to analyse and visualize next-generation sequencing data. For each file, the reads were trimmed, followed by *in silico* ribodepletion by aligning reads to a *S. mutans* UA159 rRNA gene file. The unaligned reads for each sample were then collected in a new file and used for the rest of the analysis pipeline. These reads were subjected to the ‘RNA-Seq analysis’ function following the recommended CLC Genomics Workbench pipeline for prokaryotes, using the *S. mutans* UA159 genome (NCBI Reference Sequence NC_004350.2) for aligning reads. Differential expression (DE) analysis was then performed on these aligned read files, using the ‘Differential Expression for RNA-Seq’ function, which employs trimmed mean of M values normalization and multi-factorial statistics based on a negative binomial general linearized model. Three DE comparisons were performed: LSMMG late-exponential/LSMMG mid-exponential, NG late-exponential/NG mid-exponential and 0 g RPM late-exponential/0 g RPM mid-exponential. The decision to compare gene expression changes within each model, and not across different models at comparable growth phases, was driven by two main considerations: [1] Late-exponential phase 0 g RPM cultures had modest but statistically significant increases in c.f.u. ml^−1^ and OD_600_ values, as well as decreased culture pH values, compared to NG and LSMMG cultures (Fig. 2), complicating the interpretation of comparing gene expression changes across models at this growth phase, and [2] lack of an appropriate 1 g control for the RPM that accounts for variables such as HARV movement and instrument vibrations. For each DE comparison, the following cutoffs and statistics were applied: a minimum average counts per million ≥10 for each gene in both samples, minimum average 80% unique mapped reads for each gene in both samples, ≥2 fold change (FC), false discovery rate (FDR)-adjusted *P*-value ≤0.05 [Wald test, with Benjamini–Hochberg calculation FDR=0.1 [16]]. FASTQ files and metadata for this study are available through NASA’s Open Science Data Repository (https://osdr.nasa.gov/bio/repo/data/studies/OSD-987).

**Fig. 2.**
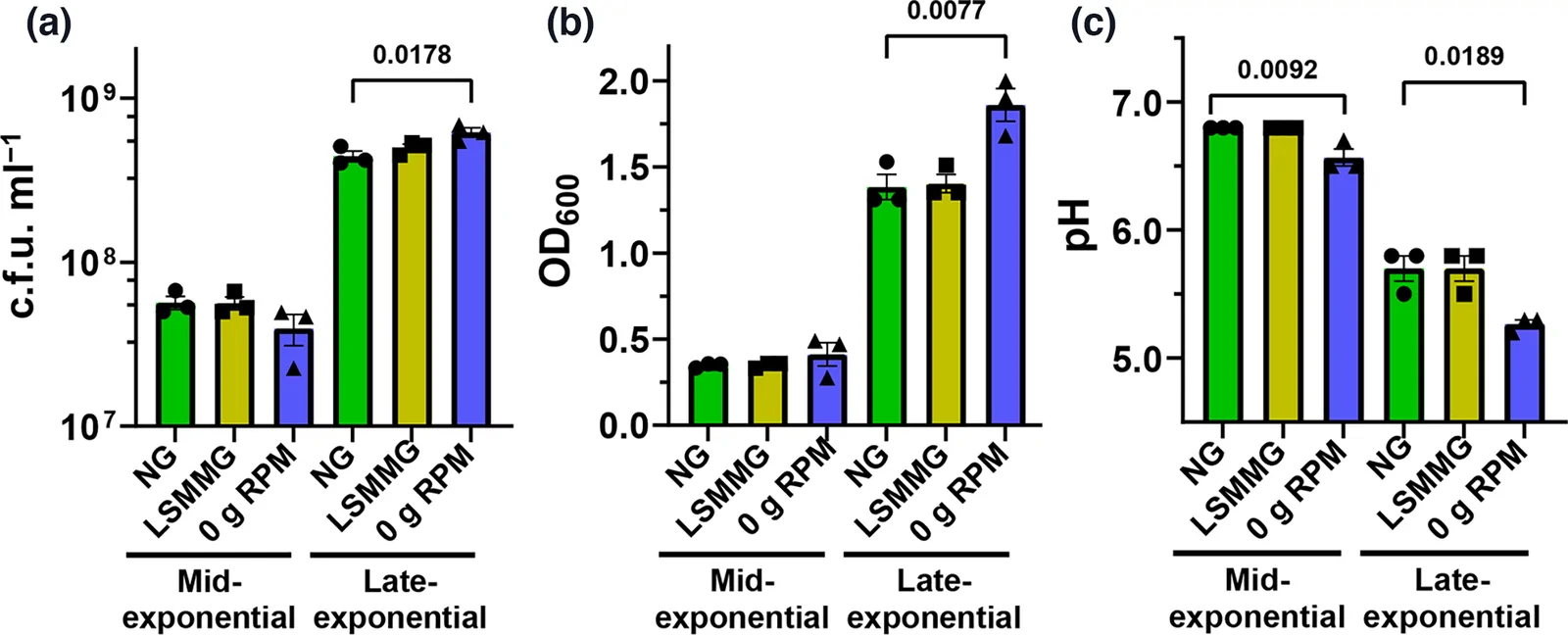
c.f.u. ml^−1^ (a), OD_600_ (b) and pH (c) growth data corresponding to mid-exponential and late-exponential growth-phase RNA-Seq experiments. Overnight *S. mutans* UA159 cultures were diluted to an OD_600_=0.05 in BM containing 11 mM glucose and 10 mM sucrose. Diluted inocula were used to fill disposable 50 ml HARVs, which were grown to mid-exponential or late-exponential growth phase at 37 °C, 5% CO_2_ in the RCCS system in NG (green) or LSMMG (simulated microgravity, yellow) orientations, as well as in HARVs attached to an RPM (blue) on ‘partial g mode’ set for 0 g. Data represents (*n*=3) biological replicates. Statistics were performed on all samples in each growth phase using one-way ANOVA followed by Dunnett’s test (compared to NG reference group). *P*-values of significant differences are indicated.

### Bioinformatics and statistical analyses

Bioinformatics tools used to analyse DE data included CLC Genomics Workbench 25 for principal component analysis (PCA), Venny 2.1 for Venn diagrams [17], PaintOmics 4 [18] for KEGG pathway enrichments, ShinyGo 0.80 for GO term enrichments [19] and UniProt for gene functional annotations [20]. GraphPad Prism 11 (and associated statistical analysis, as indicated) was used for visualization of PCA, c.f.u., OD and pH data.

## Results

### *S. mutans* growth properties in RCCS and 0 g RPM cultures

At both the mid-exponential and late-exponential growth phases, *S. mutans* growth characteristics (c.f.u. ml^−1^, OD_600_ and pH values) were similar across both the RCCS (LSMMG and NG orientations) and the 0 g RPM cultures, except for the late-exponential phase 0 g RPM cultures, which had a modest biological but statistically significant increase in c.f.u. ml^−1^ and OD_600_ values compared to NG cultures (Fig. 2a, b). Slightly increased growth of the late-exponential phase 0 g RPM culture was also indicated by its lower pH relative to the NG and LSMMG cultures (Fig. 2c). Considering these subtle growth differences, DE analysis of the corresponding RNA-Seq data focused on comparing gene expression changes between the later and earlier time points within each model (NG, LSMMG and 0 g RPM), as opposed to direct comparison of the different models at each time point.

### Altered cell clumping is a conserved phenotype between RCCS LSMMG and 0 g RPM microgravity models

In our previously published study [7], *S. mutans* LSMMG RCCS cultures exhibited 3D cell aggregates that were distinct from those observed in the NG control cultures. Specifically, early stationary-phase cells from NG RCCS cultures formed loosely packed, large aggregates of cells that tended to form flat mats close to the surface of the oxygen-permeable membrane, whereas LSMMG-grown cells formed compact spherical aggregates that were more difficult to disrupt compared to the NG cell clumps [7]. This altered cell clumping phenotype was also observed in both the LSMMG RCCS and 0 g RPM models at mid-exponential and late-exponential growth phases, but not in the NG cultures (Fig. 3), suggesting that this phenotype is a conserved feature of the response of *S. mutans* to simulated microgravity.

**Fig. 3.**
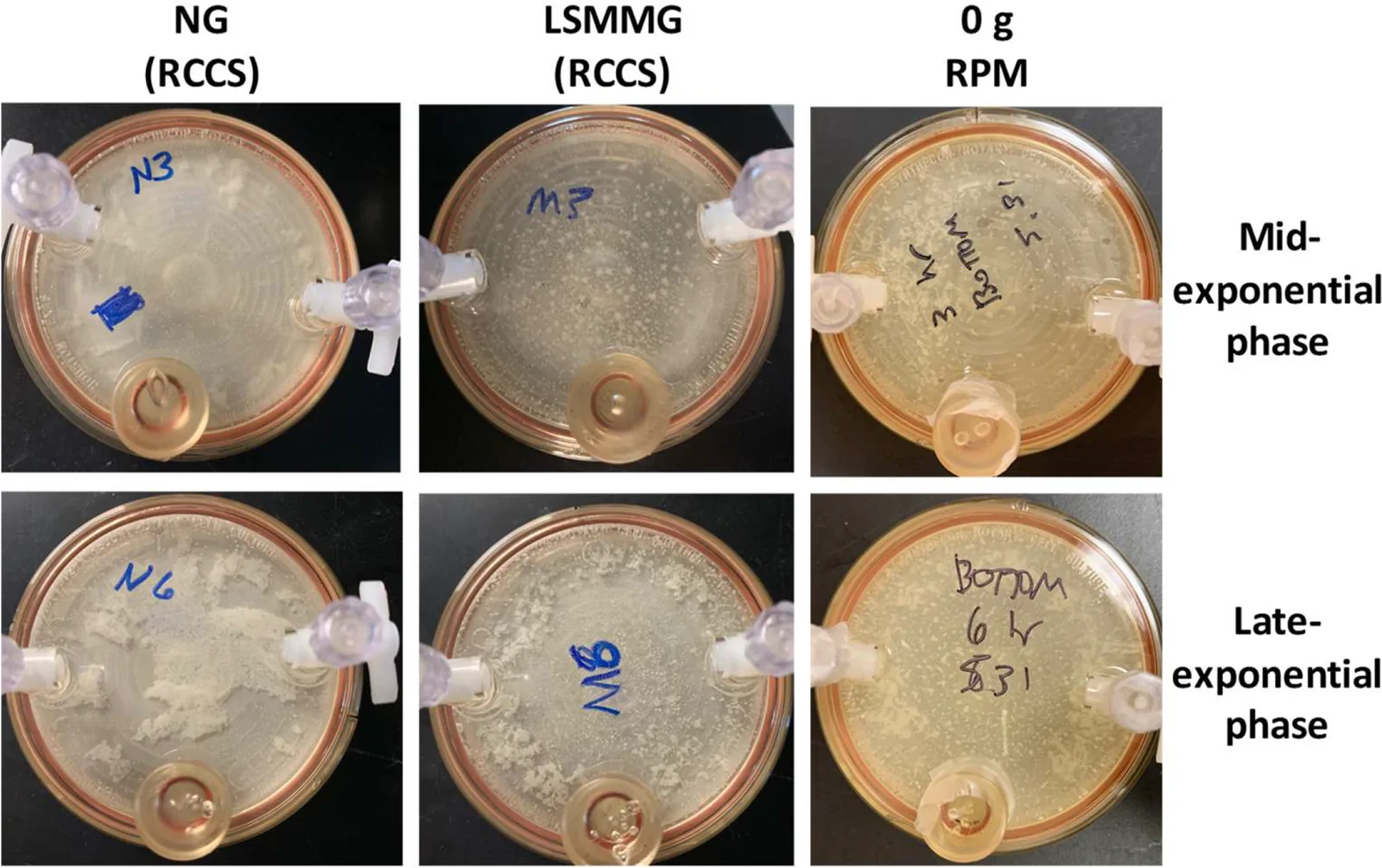
Cell clumping phenotypes in RCCS simulated microgravity (LSMMG), RCCS NG and RPM on ‘partial G motion mode’ set for 0 g. Overnight *S. mutans* UA159 cultures were diluted to an OD_600_=0.05 in BM containing 11 mM glucose and 10 mM sucrose. Diluted inocula were used to fill disposable 50 ml HARVs, which were grown to mid-exponential (3–3.5 h) or late-exponential (6–6.25 h) growth phase at 37 °C, 5% CO_2_. Cell clumping was documented prior to cell harvest and processing as described in Fig. 1. Data are representative of at least *N*=3 biological replicates.

### DE analysis of growth-phase-dependent gene expression changes in LSMMG, NG and 0 g RPM cultures

PCA of all RNA-Seq samples (Fig. 4) suggested that 29.8% of the variability between samples (PC 1) was driven by growth phase (mid-exponential vs. late-exponential), which was particularly apparent for the LSMMG and NG samples. Variability along PC 2 (26.0%) appeared to be driven primarily by growth model (0 g RPM, LSMMG and NG), which was especially obvious in the late-exponential phase samples. Overall, a large degree of variability was observed on the PCA plot between the mid-exponential phase biological replicates for all growth conditions (LSMMG, NG and 0 g RPM). This was also confirmed by heat map hierarchical clustering analysis (Fig. S1A, available in the online Supplementary Material), which showed that mid-exponential phase LSMMG and NG samples tended to cluster by experimental replicates as opposed to growth model. It is unclear if this was due to technical and/or experimental variability, or possibly transcriptome changes are not heavily influenced by simulated microgravity this early in the growth phase. Based on these results, no outliers were identified or excluded from subsequent analysis. Increased separation and distinct clustering by growth model (0 g RPM, LSMMG and NG) was observed in the late-exponential phase samples (Figs 4 and S1B).

**Fig. 4.**
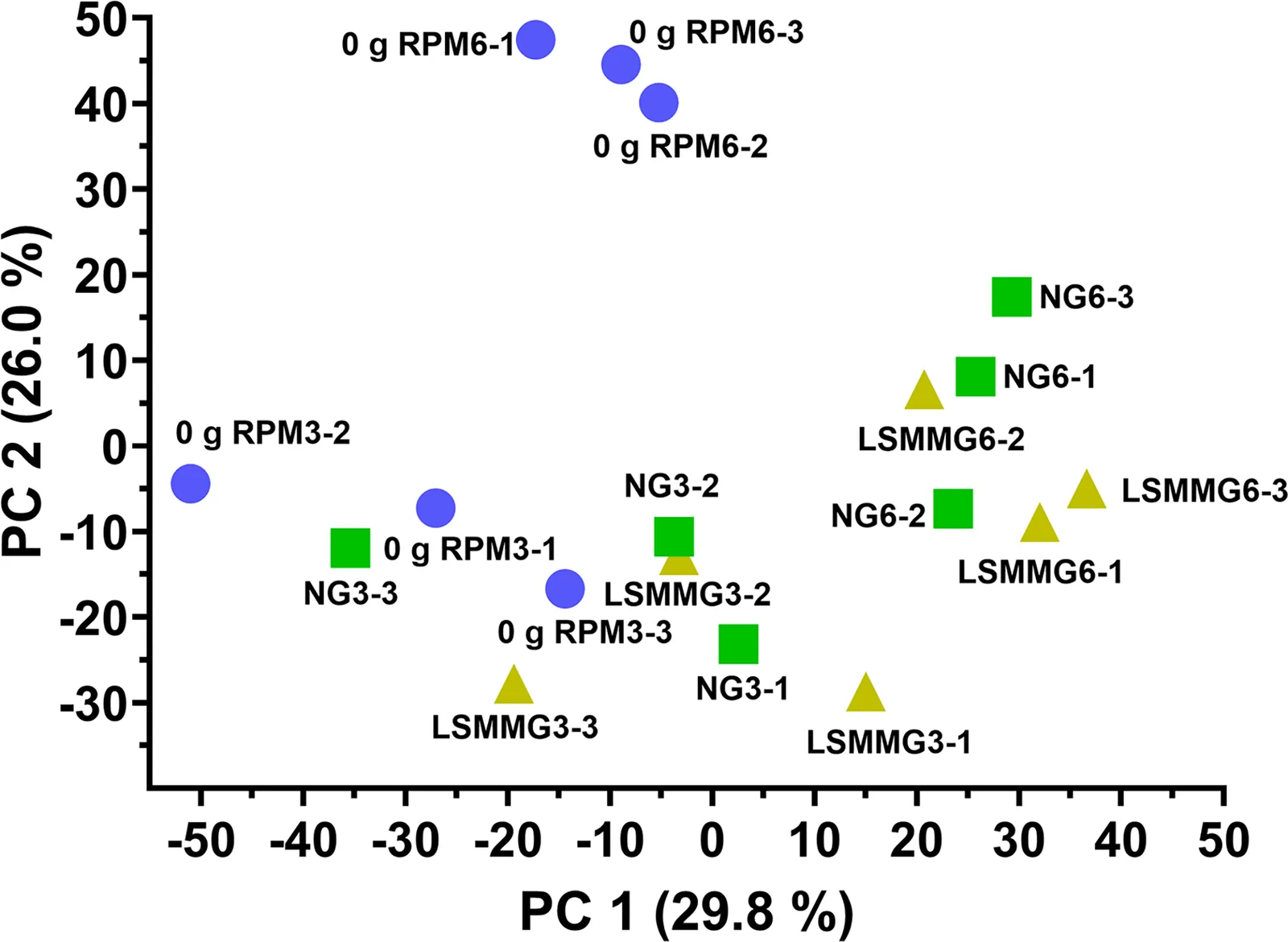
PCA plots of mid-exponential and late-exponential growth RNA-Seq data. RNA was isolated from (*n*=3) biological replicates each of HARV cultures grown in the RCCS system in LSMMG (yellow triangle) and NG (green square) orientations, as well as in HARVs attached to an RPM on ‘partial g motion mode’ set for 0 g (blue circle). Biological replicates are numbered 3-1 through 3-3 and 6-1 through 6-3 for mid-exponential and late-exponential phase samples, respectively, for each experimental group.

After application of the cutoff criteria described in Methods, a total of 252 DE genes (152 with increased and 100 with decreased expression) were observed in the late-exponential phase relative to mid-exponential phase in 0 g RPM cultures (Fig. 5). The most highly upregulated genes included sugar transport [phosphotransferase system (PTS)] subunits and pyrimidine biosynthesis enzymes, whereas the most strongly downregulated genes encoded a variety of hypothetical proteins and competence-related proteins (Table S1). Fewer total DE genes (late-exponential phase/mid-exponential phase) were observed in the LSMMG (161 total; 82 with increased and 79 with decreased expression) and NG growth conditions (159 total; 91 with increased and 68 with decreased expression) (Fig. 5). For LSMMG, the most highly upregulated genes included TCA cycle enzymes and ABC transporters, whereas the most strongly downregulated genes included members of the TnSMU2-encoded non-ribosomal peptide synthetase–polyketide synthase gene cluster and competence genes (Table S2). For NG, the most highly upregulated genes included carbohydrate transport/metabolism and pyruvate import, whereas the most strongly downregulated genes were the same as observed in LSMMG (TnSMU2 genes, competence genes) (Table S3), suggesting that downregulation of these genes is a growth-phase transition specific to the RCCS growth model and not a simulated microgravity-specific response.

**Fig. 5.**
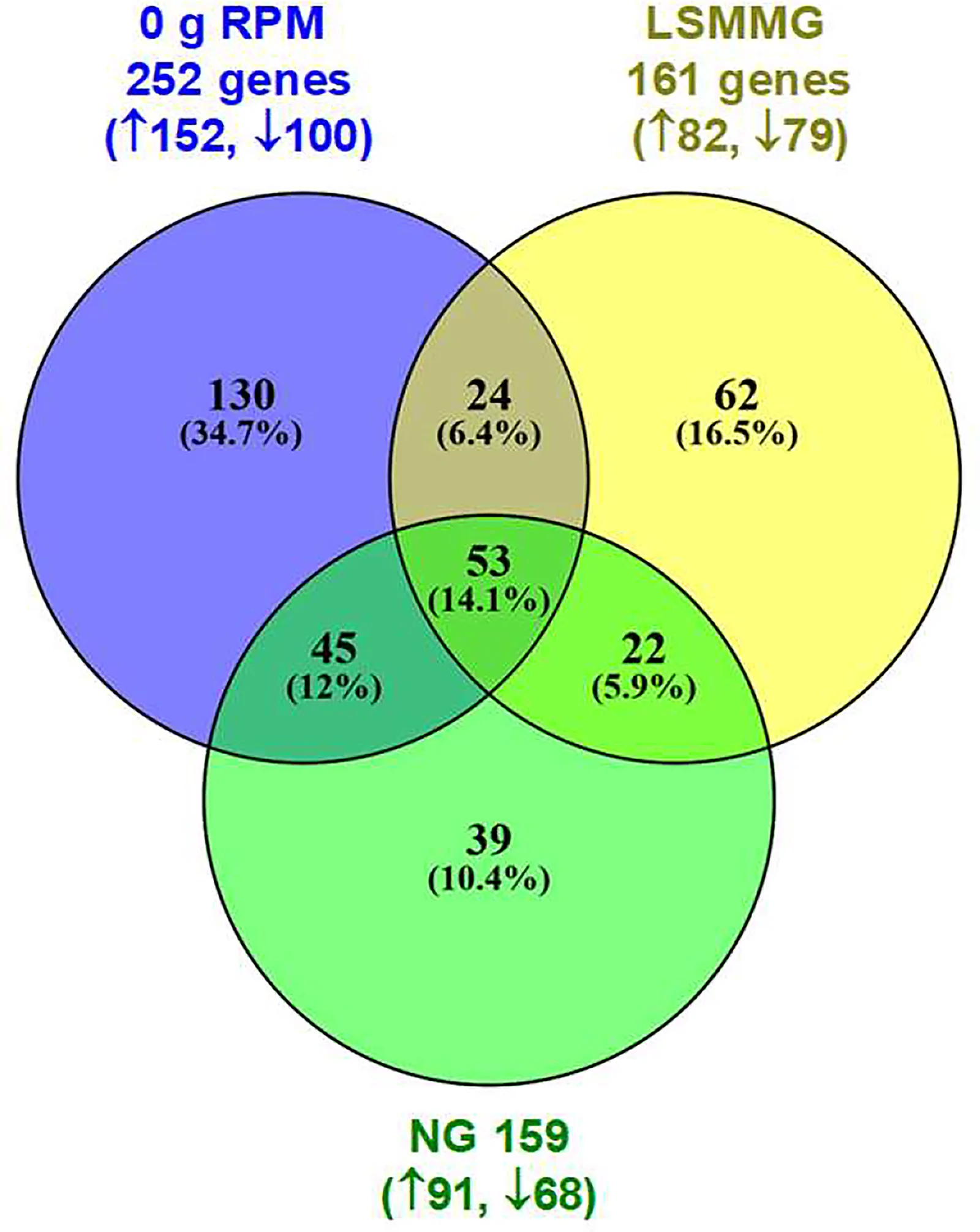
Venn diagram of differentially expressed *S. mutans* genes in each growth condition. Genes with significant DE comparing LSMMG late-exponential/LSMMG mid-exponential (yellow), NG late-exponential/NG mid-exponential (green) and 0 g RPM late-exponential/0 g RPM mid-exponential (blue). Venn includes genes with an FC of >2.0 and FDR-adjusted *P*-value <0.05. The number of up- and downregulated genes is indicated by arrows. Analysis performed with Venny 2.1.

### Growth-phase-dependent functional enrichments in LSMMG, NG and 0 g RPM cultures

PaintOmics analysis of DE expressed genes in LSMMG, NG and 0 g RPM cultures revealed unique significant (*P*<0.1) KEGG metabolic pathway enrichments in each growth model (Table 1). *P*-values <0.1 were chosen as an exploratory cutoff in the context of comparing biological trends and metabolic pathways shared between more than one growth condition (LSMMG, NG and 0 g RPM). LSMMG exhibited the greatest number of unique pathway enrichments (purine metabolism, cofactor biosynthesis, arginine biosynthesis, one-carbon fixation, 2-oxocarboxylic acid and styrene degradation), whereas both NG (lipoic acid metabolism, ABC transporters, two-component systems) and 0 g RPM (galactose metabolism, oxidative phosphorylation and fructose/mannose metabolism) each contained three unique enrichments. Interestingly, overlapping enriched pathways were also identified, with TCA cycle and folate metabolism pathways shared between NG and LSMMG, sucrose and pyrimidine metabolism shared between 0 g RPM and LSMMG, PTS, amino/nt sugar metabolism and tRNA biosynthesis shared between 0 g RPM and NG and folate biosynthesis common to all three growth conditions. Shared enrichments of the TCA cycle and folate metabolism (between LSMMG and NG) and folate biosynthesis (between all three growth conditions) suggest that these pathways represent growth-phase transition specific changes that are not related to simulated microgravity growth. GO-term enrichment analysis (ShinyGo) also confirmed many of the metabolic pathway enrichments in all three growth models (Fig. S2).

**Table 1.** Summary of enriched KEGG metabolic pathways in 0 g RPM, LSMMG and NG growth conditions

Pathway name	Unique gene			*P*-value		
	**0 g RPM**	**LSMMG**	**NG**	**0 g RPM**	**LSMMG**	**NG**
Purine metabolism	29	29	29	0.129	0.00017	0.279
Citrate cycle (TCA cycle)	10	10	10	0.26	0.0109	0.0093
Folate biosynthesis	11	11	11	0.032	0.0159	0.014
Starch and sucrose metabolism	29	30	31	0.022	0.061	0.151
One-carbon pool by folate	10	10	10	0.26	0.063	0.0093
Biosynthesis of cofactors	54	54	55	0.37	0.066	0.901
Pyrimidine metabolism	24	24	24	0.049	0.073	0.908
Arginine biosynthesis	11	11	11	1.00	0.082	1.00
Other carbon fixation pathways	11	11	11	1.00	0.082	1.00
2-Oxocarboxylic acid metabolism	25	25	25	0.857	0.085	0.195
Styrene degradation	1	1	1	0.179	0.097	1.00
PTS	24	26	26	0.0012	0.933	0.0067
Galactose metabolism	14	14	14	0.025	1.00	0.133
Amino sugar and nt sugar metabolism	27	27	27	0.037	0.499	0.095
Aminoacyl-tRNA biosynthesis	19	19	19	0.038	0.278	0.09
Oxidative phosphorylation	8	9	7	0.038	1.00	1.00
Fructose and mannose metabolism	21	21	21	0.064	1.00	0.121
Lipoic acid metabolism	7	7	7	0.113	0.513	0.0019
ABC transporters	94	97	95	0.145	0.334	0.001
Two-component system	42	44	43	0.332	0.824	0.092

Enriched KEGG pathways with raw *P*-values <0.1 for each growth condition are shown. Grey, pathway enriched in all three growth conditions; green, pathway enriched in LSMMG and NG; orange, pathway enriched in 0 g RPM and LSMMG; blue, pathway enriched in 0 g RPM and NG. Analysis performed with PaintOmics 4.

### Identification of *S. mutans* shared simulated microgravity regulon

Venn analysis of all three comparisons (Fig. 5) revealed an overlap of 24 DE genes common to both LSMMG and 0 g RPM simulated microgravity models (listed in Table 2). Shared upregulated late-exponential phase expression genes were involved in uridine monophosphate (UMP) and pyrimidine biosynthesis, ferrous iron transport and translation. Downregulated genes common to all three comparisons included purine biosynthesis, malate metabolism, serine biosynthesis and transcription regulation. Expression of *glgA* (glycogen synthase) was upregulated in the late-exponential phase 0 g RPM cultures but downregulated in LSMMG cultures (Table 2). *S. mutans* GlgA promotes intracellular glycogen storage, which can be used as a carbohydrate source to fuel fermentation and prolong acid production upon nutrient depletion [21–23]. Therefore, it is possible that the increased growth yields and decreased culture pH of late-exponential phase 0 g RPM cultures are related to increased intracellular glycogen storage compared to LSMMG and NG cultures. Many of these overlapped gene expression changes between LSMMG and 0 g RPM cultures were also reflected in the KEGG pathway and GO-term enrichment data (Table 1 and Fig.S2, respectively).

**Table 2.** Gene expression changes common to 0 g RPM and LSMMG growth conditions All FC values represent late-exponential (LE) growth/mid-exponential (ME) growth under the indicated growth condition.

Gene	LE/ME FC (0 g RPM)	LE/ME FC (LSMMG)	Function	Gene Ontology
*pyrDB*	8.4	2.8	Dihydroorotate dehydrogenase (DHOD) (DHODase)	*De novo* pyrimidine nucleobase biosynthetic process (GO:0006207); *de novo* UMP biosynthetic process (GO:0044205)
*SMU_857*	8.1	2.2	Uracil permease	
*pyrK*	8	2.6	Dihydroorotate dehydrogenase B [NAD(+)], electron transfer subunit (dihydroorotate oxidase B)	
*pyrE*	4.1	2.2	Orotate phosphoribosyltransferase (OPRT)	
*pyrF*	3.6	2	Orotidine 5′-phosphate decarboxylase (OMP decarboxylase) (OMPDCase)	
glgA*	3.4	−2.8	Glycogen synthase (starch [bacterial glycogen] synthase)	Glycogen biosynthetic process (GO:0005978)
*SMU_1219* c	3	2.2	MmcQ-like protein	
*SMU_774*	2.8	2.2	Hydrolase	
*nylA*	2.5	2.1	Amidase	6-Aminohexanoate-cyclic-dimer hydrolase activity (GO:0019874)
*SMU_541*	2.4	2.5	DUF910 family protein	
*SMU_170*	2.2	2	Small ribosomal subunit protein uS9 (30S ribosomal protein S9)	Translation (GO:0006412)
*SMU_1351*	2.2	2.2	Transposase	
*feoB*	−2	−2.1	Ferrous iron transport protein B	Ferrous iron transmembrane transporter activity (GO:0015093); transition metal ion binding (GO:0046914)
*feoA*	−3.5	−2.4	Ferrous ion transport protein A	
*purL*	−3.1	−4.7	Phosphoribosylformylglycinamidine synthase (FGAM synthase)	Purine nt biosynthetic process (GO:0006164); *de novo* inosine monophosphate (IMP) biosynthetic process (GO:0006189)
*purF*	−3.1	−5.1	Amidophosphoribosyltransferase (ATase) (glutamine phosphoribosylpyrophosphate amidotransferase) (GPATase)	
*SMU_31*	−3.5	−4.6	Phosphomethylpyrimidine kinase	
*SMU_29*	−4.9	−4	Phosphoribosylaminoimidazole-succinocarboxamide synthase (SAICAR synthetase)	
*mleS*	−3.3	−2.6	Malolactic enzyme	Malate metabolic process (GO:0006108); malolactic fermentation (GO:0043464)
*SMU_138*	−3.1	−2.3	Malate permease	
*serC*	−2.1	−2.1	Phosphoserine aminotransferase (phosphohydroxythreonine aminotransferase) (PSAT)	l-Serine biosynthetic process (GO:0006564); pyridoxine biosynthetic process (GO:0008615)
*SMU_2035*	−2.1	−2	ld-Carboxypeptidase	Hydrolase activity (GO:0016787)
*SMU_1072* c	−2.6	−3.7	Acetyltransferase	Acyltransferase activity, transferring groups other than amino-acyl groups (GO:0016747)
*SMU_136* c	−3.8	−2	Transcriptional regulator	DNA binding (GO:0003677); DNA-binding transcription factor activity (GO:0003700)

*Altered *glgA* expression in the late-exponential phase is shared between 0 g RPM and LSMMG models but displays opposite patterns of regulation, likely due to LE growth differences in these two models (see results).

## Discussion

Overall, these transcriptomic results suggest a small but reproducible overlap in *S. mutans* growth-phase-dependent gene expression changes between 0 g RPM and LSMMG-simulated microgravity cultures, when grown in sucrose-containing medium that promotes biofilm formation. This relatively small overlap aligns with studies comparing previously published bacterial transcriptome data from both spaceflight and simulated microgravity experiments [11, 12], which revealed that none of the spaceflight and LSMMG responses appeared to be consistent across datasets between these models and/or the species of bacteria under study. Interestingly, the mid-exponential biological replicate transcriptome samples within each experimental group of our study (LSMMG, NG and 0 g RPM) did not display distinct PCA clusters compared to the late-exponential phase samples (Fig. 4). Therefore, it is possible that transcriptomic differences that occur in response to simulated microgravity may not be apparent until later phases of growth, and/or perhaps simulated microgravity has a more profound effect on other aspects of bacterial physiology (i.e. alterations in the proteome) during exponential growth. Given the lack of published gene expression studies of *S. mutans* during growth in low-Earth orbit, it is difficult to predict which of the LSMMG or 0 g RPM ground-based simulated microgravity models may better reflect the impact of true microgravity on the *S. mutans* transcriptome. Additionally, the transcriptome data presented herein and previously by our research group [7] represents a short-term (hours) acclimation response of *S. mutans* to simulated microgravity, whereas other studies have investigated the mid-exponential phase transcriptome response of *S. mutans* populations previously evolved for 100 days under NG or LSMMG using the RCCS system [24], providing insights into the long-term adaptive *S. mutans* transcriptome response to simulated microgravity. Interestingly, distinct metabolic and GO-term enriched pathways were observed in both of our LSMMG and 0 g RPM late-exponential (LE)/mid-exponential (ME) datasets (starch and sucrose metabolism, pyrimidine metabolism) compared to the GO-term enriched pathways uniquely identified in the transcriptomes of long-term LSMMG-adapted cultures [24], which included strongly upregulated lactose catabolic genes and downregulated mutanobactin genes. These data suggest that *S. mutans* may undergo distinct short-term and long-term transcriptome responses to simulated microgravity.

DE genes (LE phase/ME phase) and/or enriched metabolic pathways related to pyrimidine metabolism were common to both LSMMG and 0 g RPM growth models, with expression of these genes upregulated in the late-exponential growth phase. Interestingly, our previously published *S. mutans* 8-h LSMMG experiment [7], which was grown in the same medium and RCCS system as this current study, demonstrated increased cellular levels of pyrimidine intermediates (UMP, uracil) in LSMMG cells relative to NG cells, and pyrimidine deoxyribonucleotide biosynthesis gene expression was significantly downregulated in the corresponding transcriptomic data [7]. There appears to be an emerging link between altered pyrimidine metabolism and exposure to simulated microgravity. For example, KEGG pathway analysis of the gut microbiota changes in humans exposed to simulated microgravity (diamagnetic material magnetic levitation method) showed a predicted decrease in microbial pyrimidine metabolism [25]. Conversely, metabolite profiling of the caecal contents of rats exposed to simulated weightlessness using a hindlimb unloading model demonstrated a significant increase in metabolites involved in pyrimidine metabolism, which correlated with changes in intestinal microbiota and immunological characteristics [26]. *Bacillus subtilis* transcriptome DE (LSMMG/NG) data also revealed that pyrimidine metabolism was an upregulated and significantly enriched KEGG pathway when grown in LSMMG [12]. Pyrimidine pathways have central metabolic importance in DNA and RNA synthesis, management of cellular energy and growth regulation (reviewed in [27]). Disruption of *Staphylococcus aureus de novo* pyrimidine biosynthesis resulted in altered Sae-dependent virulence gene expression and cell envelope defects under pyrimidine-limited conditions [28], whereas a thymidylate synthase (*thyA*) mutant displayed increased expression of pyrimidine biosynthesis genes, decreased expression of global virulence gene regulators and decreased virulence in both *Caenorhabditis elegans* killing and murine pneumonia models of infection [29]. Furthermore, decreased capsule expression was observed in a *Streptococcus pneumoniae* pyrimidine biosynthesis mutant [28–30]. Collectively, these studies suggest that both overexpression and disruption of pyrimidine biosynthesis could have effects on the virulence potential of bacterial pathogens. Given that the functional consequences of upregulated pyrimidine biosynthesis on the virulence potential of oral streptococci remain to be determined, the mechanistic link between altered pyrimidine gene expression in simulated microgravity will be further elucidated in our ongoing and future studies, with specific focus on how this aspect of metabolism impacts *S. mutans* stress resistance and virulence potential.

Current research indicates that the environment of spaceflight or an environment simulating spaceflight promotes changes in virulence-associated microbial characteristics, gene expression or biofilm formation [31–34]. For instance, compared to ground control, *Salmonella* spp. cultured in the ISS displayed elevated gene expression linked to biofilm formation, including the *wca*/*wza*, *ompA* and *fim* genes [33]. In comparison to terrestrial controls, *Candida albicans*, a commensal yeast and a frequent cause of fungal infection, demonstrated greater cell aggregation, increased cell proliferation and resistance to the antifungal amphotericin B [35, 36]. *S. mutans* LSMMG and 0 g RPM cultures displayed a conserved altered cell clumping phenotype (Fig. 3) that was also previously observed in early stationary-phase LSMMG studies of *S. mutans* [7]. In both studies, sucrose-dependent cell clumping was used as a proxy for biofilm-forming potential in the LSMMG, 0 g RPM and NG models, as it is difficult to accurately assess biofilm structure in HARVs at the microscopic level without disturbing the structure of the clumps. Sucrose-dependent (glucosyltransferase, glucan-binding protein) biofilm genes were upregulated at the LE phase in all three growth conditions (NG, LSMMG and 0 g RPM), suggesting that post-transcriptional or protein-level modifications may be responsible for the *S. mutans* altered clumping phenotype observed in simulated microgravity.

Astronauts need good oral health before they embark on spaceflight missions, especially in the context of long-term journey to the moon and beyond [5, 37]. The effects of space travel and diet on human dental health were originally assessed in Skylab missions, the first US space station launched by NASA that was occupied by astronauts for 24 weeks [5]. This research revealed that astronauts returned to Earth with higher levels of bacteria in their oral cavity, including *Bacteroides*, *Veillonella*, *Fusobacterium*, *Neisseria*, *S. sanguinis* and *S. mutans* [5]. Gravity has a very basic impact on biological systems since life has developed under its continual influence. Therefore, furthering our understanding of *S. mutans* physiology and biofilm responses in simulated microgravity models will refine our predictions on how this bacterium will respond to space travel conditions.

## Supplementary material

10.1099/mic.0.001776 Supplementary Material 1.

## Abbreviations

BHI: brain heart infusion
BM: biofilm medium
DE: differential expression
FC: fold change
FDR: false discovery rate
HARV: high-aspect rotating vessel
ISS: International Space Station
LSMMG: low-shear modelled microgravity
NG: normal gravity
PCA: principal component analysis
PTS: phosphotransferase system
RCCS: rotary cell culture system
RIN: RNA integrity number
RPM: random positioning machine
