# Supplementary material for: Comparison of Streptococcus mutans growth-phase-dependent transcriptome changes in two different simulated microgravity models
Source: Microbiology (Reading). 2026 Oct 5;172(10):001776. doi: 10.1099/mic.0.001776 (PMC13637945; doi:10.1099/mic.0.001776)
Supplement: Supplementary file 1 [file mic-172-01776-s001.pdf]

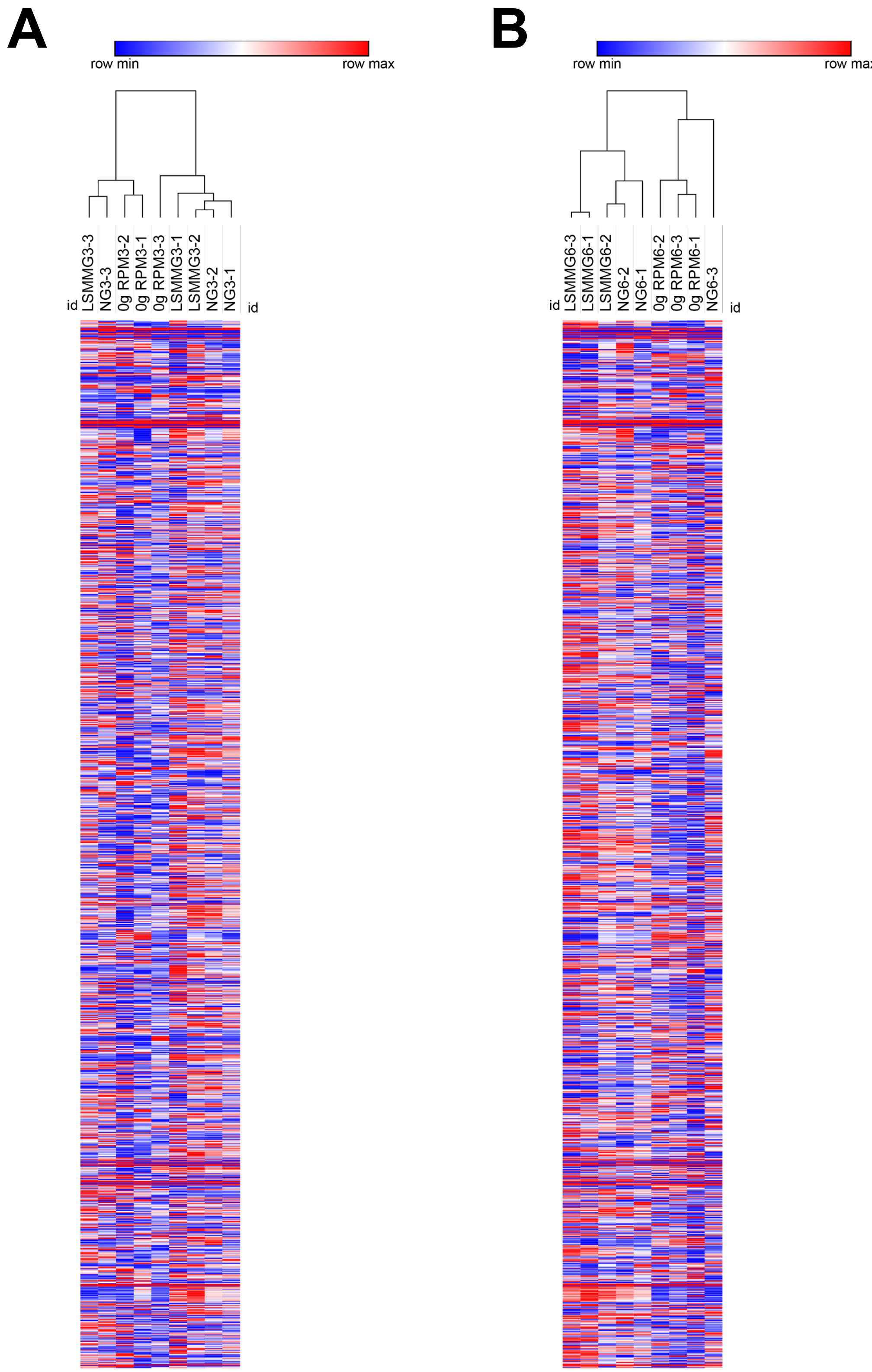

Supplemental Figure S1. Heat maps of mid-exponential phase (A) and late-exponential phase (B) transcriptomes. Heat maps were generated with Morpheus (https://software.broadinstitute.org/morpheus), using TMM-normalized counts per million (CPM) values for all genes (no cutoffs applied to data) in LSMMG, NG, and 0 g RPM datasets. Samples are clustered based on similarity calculated by 1 – Pearson correlation and average linkage method (indicated by tree at the top of the heat map).

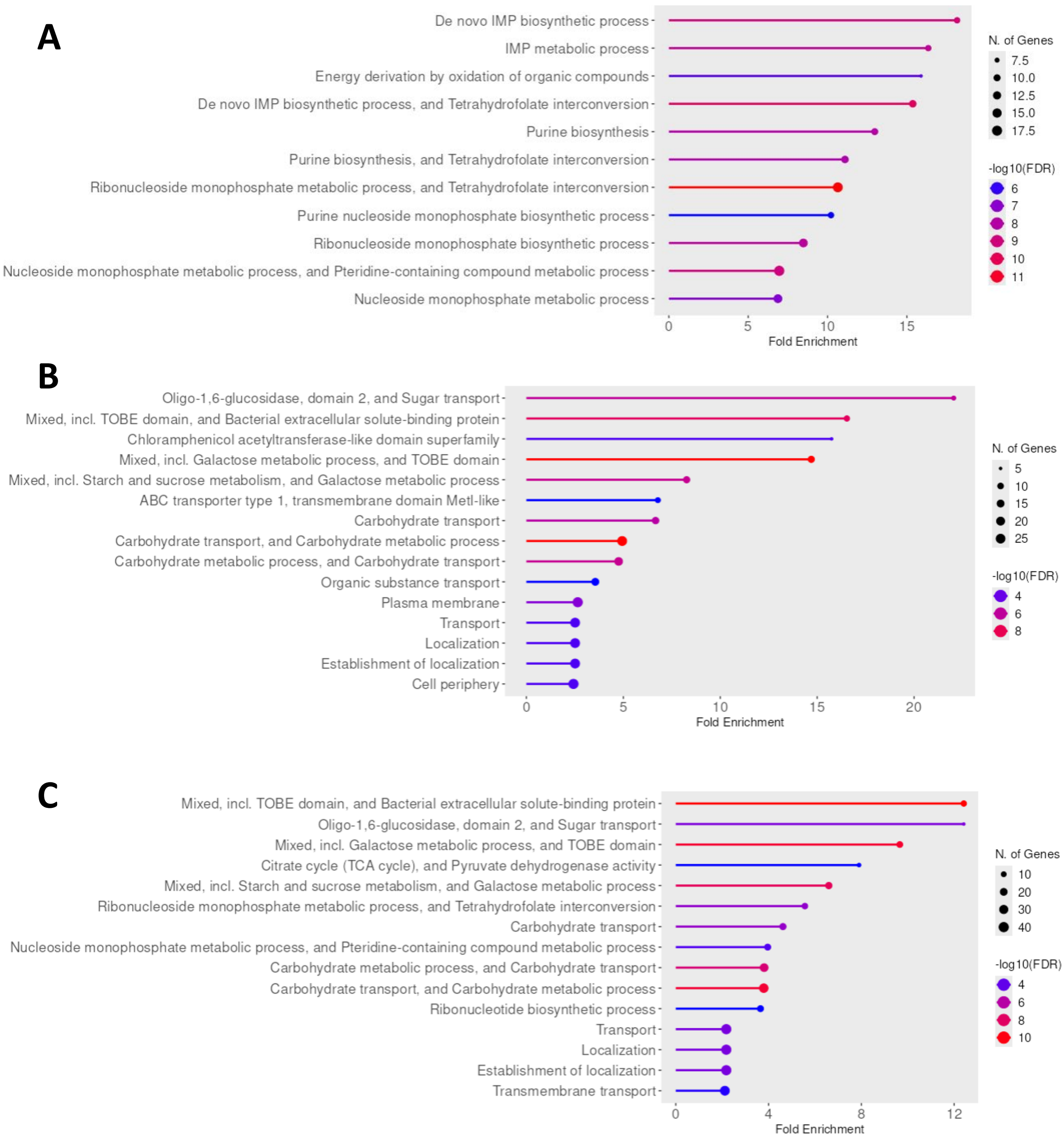

Supplemental Figure S2. Functional enrichments of LSMMG (A), NG (B) and 0 g RPM (C) gene expression changes (late-exponential/mid-exponential growth). Top 15 significant enrichments (FDR cutoff < 0.05). ShinyGo 0.85.

Supplemental Table S1. Summary of RNA-Seq read counts and mapping rates.

SAMPLE	Initial read count (million)	Post-in silico ribodepletion read count (million)	Mapping rate (%)
LSMMG3-1	45.3	41.5	98.8
LSMMG3-2	52.4	42.6	98.5
LSMMG3-3	49.5	42.4	98.8
NG3-1	51.4	42.2	98.4
NG3-2	50.6	38.1	98.1
NG3-3	43.5	35.9	97.5
0 g RPM3-1	43.1	34.4	98.0
0 g RPM3-2	40.8	25.1	98.1
0 g RPM3-3	56.0	41.2	98.6
LSMMG6-1	44.7	39.8	98.5
LSMMG6-2	47.6	41.1	98.3
LSMMG6-3	57.7	52.6	98.7
NG6-1	53.3	46.6	98.6
NG6-2	59.9	52.8	98.7
NG6-3	50.6	44.9	98.7
0 g RPM6-1	36.3	24.4	97.7
0 g RPM6-2	57.7	44.4	97.2
0 g RPM6-3	45.4	33.1	97.3

Supplemental Table S2. Significant (FDR-adjusted p-value < 0.05) gene expression changes in 0 g RPM growth condition. All fold-change (FC) values represent late-exponential (LE) growth/mid-exponential (ME) growth during 0 g RPM growth. CPM = Counts per million (TMM-normalized).

Gene Name	0 g RPM Fold change (LE/ME)	0 g RPM log2 Fold change (LE/ME)	FDR p-value	Average CPM (LE)	Average CPM (ME)
SMU_1956c	15.08	3.91	7.7E-11	915.35	61.84
SMU_104	14.71	3.88	5.8E-10	5976.55	409.97
SMU_1957	14.65	3.87	3.07E-09	3015.42	209.55
SMU_105	11.64	3.54	1.89E-12	2092.02	180.64
SMU_1958c	10.94	3.45	7.3E-07	2117.66	196.99
pyrB	10.84	3.44	1.53E-27	1098.75	102.27
SMU_1960c	10.67	3.42	4.63E-06	951.81	91.07
SMU_1961c	10.55	3.40	1.28E-05	362.89	35.07
pyrA	9.68	3.28	2.95E-27	1378.73	143.82
SMU_103	9.68	3.27	7.3E-07	711.78	74.50
dexB	9.04	3.18	4.34E-15	437.04	48.48
msmK	8.58	3.10	1.76E-09	246.41	28.69
SMU_1914c	8.55	3.10	4.69E-10	686.40	81.29
msmF	8.38	3.07	4.03E-10	235.32	28.23
pyrDB	8.36	3.06	8.07E-21	695.90	83.73
gtfA	8.15	3.03	4.21E-10	283.56	34.80
SMU_857	8.09	3.02	2.09E-22	1414.79	176.18
SMU_1906c	8.06	3.01	1.39E-07	378.46	47.41
SMU_1908c	8.04	3.01	1.64E-07	690.82	87.20
pyrAB	8.02	3.00	3.38E-20	4523.46	571.15
pyrK	7.97	3.00	4.04E-16	369.83	46.63
msmG	7.90	2.98	5.37E-09	140.57	17.83
SMU_102	7.52	2.91	5.19E-05	2132.86	287.52
SMU_1910c	7.16	2.84	1.15E-09	1981.26	280.10
SMU_1909c	6.80	2.77	6.5E-09	1457.57	217.08
pfl	6.49	2.70	0.000385	17505.01	2721.81
SMU_151	6.03	2.59	2.33E-09	141.78	23.81
SMU_101	5.98	2.58	0.000144	2198.01	373.42
pyrR	5.94	2.57	8.54E-09	286.49	48.38
SMU_1905c	5.74	2.52	5.66E-08	167.62	29.64
msmE	5.47	2.45	2.88E-05	154.02	28.24
SMU_574c	5.14	2.36	0.009599	168.90	33.13
pdhC	5.09	2.35	0.000659	179.28	35.04
SMU_1913c	4.94	2.31	2.21E-08	917.86	187.83
SMU_423	4.80	2.26	2.94E-06	79.48	16.75
pdhB	4.61	2.21	0.002896	133.39	28.83
SMU_1571	4.49	2.17	1.97E-07	177.35	39.63

naoX	4.36	2.13	4.87E-08	2356.83	544.95
gatA	4.25	2.09	1.5E-12	1480.73	351.98
adhA	4.19	2.07	0.000629	3210.75	769.99
SMU_1217c	4.16	2.06	3.34E-09	214.00	51.93
pyrE	4.13	2.05	6.35E-13	549.61	134.35
SMU_1904c	4.04	2.02	4.5E-06	1434.26	358.88
fruA	4.01	2.00	0.000648	946.26	239.28
malF	3.92	1.97	2.32E-05	163.48	42.02
agaL	3.92	1.97	0.005587	232.43	59.82
pdhA	3.90	1.96	0.035557	108.95	28.05
SMU_1220c	3.83	1.94	4.01E-08	210.40	55.46
malG	3.80	1.93	3.38E-07	65.35	17.24
SMU_575c	3.77	1.91	0.022242	86.20	23.07
malX	3.67	1.87	0.000274	667.92	184.28
pyrF	3.64	1.86	2.48E-11	606.52	167.88
SMU_1116c	3.60	1.85	1.3E-08	1484.09	418.09
phsG	3.44	1.78	0.001549	2880.93	835.74
lytS	3.44	1.78	0.000198	563.83	166.45
fruB	3.43	1.78	6.95E-05	58.98	17.42
ahpC	3.42	1.77	1.07E-06	4020.97	1187.97
glgA	3.38	1.76	0.004231	1715.07	507.15
gbpC	3.34	1.74	7.32E-06	1354.81	408.76
lytR	3.29	1.72	0.000225	160.44	49.24
atpA	3.24	1.70	8.75E-09	870.45	271.00
SMU_292	3.24	1.69	4.2E-08	49.61	15.35
galK	3.14	1.65	0.000247	59.13	18.94
atpB	3.13	1.65	2.32E-05	3983.48	1282.78
SMU_152	3.07	1.62	0.000223	76.58	25.21
murI	3.03	1.60	8.07E-10	882.37	293.80
SMU_662	2.97	1.57	2.32E-07	74.40	25.28
SMU_144c	2.97	1.57	3.54E-06	390.12	131.86
SMU_1219c	2.97	1.57	3.86E-07	72.06	24.41
gtfB	2.94	1.55	6.38E-08	861.86	297.29
SMU_525	2.88	1.53	3.67E-09	291.19	102.37
adhB	2.85	1.51	0.009421	4703.37	1665.00
glk	2.85	1.51	4.53E-05	3504.85	1247.91
SMU_1716c	2.83	1.50	7.62E-10	739.09	264.28
SMU_180	2.83	1.50	0.039814	1230.70	439.75
SMU_524	2.82	1.50	5.57E-09	222.45	79.36
SMU_774	2.82	1.49	3.59E-05	156.03	55.69
SMU_1717c	2.81	1.49	4E-09	863.04	310.56
SMU_143c	2.79	1.48	9.68E-10	664.53	239.34
trk	2.75	1.46	5.37E-09	847.92	312.34
pttB	2.74	1.46	0.000864	74.28	27.32
SMU_260	2.72	1.44	1.47E-06	287.16	106.28
msmR	2.71	1.44	0.015129	111.49	41.71

SMU_160	2.70	1.43	2.96E-08	301.19	112.63
SMU_2127	2.68	1.42	0.035521	3532.86	1333.74
atpD	2.68	1.42	0.001684	3375.04	1271.44
atpC	2.66	1.41	0.002312	1822.09	693.77
pyrG	2.64	1.40	1.69E-05	2139.12	817.99
SMU_1405c	2.63	1.39	2.26E-06	575.55	221.57
SMU_1712c	2.61	1.39	4E-09	294.56	114.06
SMU_432	2.61	1.38	0.000251	29.66	11.45
trkB	2.60	1.38	4.93E-06	537.55	209.04
treA	2.60	1.38	0.000248	64.12	24.77
SMU_1404c	2.56	1.36	0.000263	131.16	51.48
pflA	2.55	1.35	2.8E-05	1393.46	550.28
SMU_543	2.52	1.34	3.37E-05	1631.97	655.56
SMU_650	2.50	1.32	0.000267	1967.51	794.41
pacL	2.49	1.32	0.000255	3546.20	1434.88
rluB	2.49	1.31	7.22E-07	385.34	156.65
mutY	2.48	1.31	9.44E-06	277.96	112.60
nylA	2.48	1.31	7.22E-07	289.08	117.74
scnK	2.47	1.30	5.29E-06	807.29	329.77
glgP	2.46	1.30	0.001161	688.38	282.79
gatB	2.45	1.29	0.000316	1899.30	783.01
SMU_242c	2.44	1.29	0.00097	276.57	114.13
SMU_1820c	2.43	1.28	2.56E-05	1755.70	729.33
atpG	2.43	1.28	0.003191	1545.86	645.52
SMU_1511c	2.42	1.28	3.37E-05	237.08	98.75
SMU_541	2.42	1.28	0.002312	34.33	14.31
SMU_438c	2.40	1.26	0.037847	431.39	181.08
SMU_1714c	2.40	1.26	7.91E-07	236.85	99.71
scnR	2.38	1.25	3.91E-06	439.20	185.60
SMU_1487	2.38	1.25	6.04E-05	88.08	37.29
syfA	2.37	1.25	0.000251	625.67	266.21
SMU_765	2.35	1.23	0.004075	11375.33	4895.66
SMU_440	2.33	1.22	0.019338	277.24	120.62
syt1	2.33	1.22	0.0001	1138.69	492.19
SMU_161	2.31	1.20	4.31E-05	149.92	65.49
syfB	2.30	1.20	0.000198	1667.58	731.32
SMU_1715c	2.30	1.20	2.37E-07	250.20	109.63
SMU_1403c	2.29	1.20	0.001053	47.36	20.83
SMU_1719c	2.26	1.18	1.36E-06	376.94	168.78
SMU_616	2.25	1.17	0.000856	748.53	334.34
SMU_1988c	2.23	1.16	0.029145	674.06	303.82
SMU_1402c	2.21	1.14	0.007416	25.80	11.80
SMU_312	2.21	1.14	5.41E-05	55.49	25.26
adcA	2.19	1.13	8.5E-07	480.54	221.72
SMU_241c	2.19	1.13	3.07E-05	229.65	105.66
SMU_124	2.18	1.12	0.004075	852.62	395.40

SMU_2066c	2.17	1.12	0.000178	1295.87	602.65
SMU_1155	2.17	1.12	0.00097	94.72	44.09
SMU_170	2.16	1.11	0.003168	2267.06	1057.09
SMU_1900	2.16	1.11	0.000274	63.59	29.71
SMU_1351	2.16	1.11	0.000629	37.02	17.33
SMU_311	2.15	1.10	0.001714	28.02	13.08
cah	2.13	1.09	0.001741	100.89	47.84
SMU_331	2.11	1.08	6.41E-07	538.18	257.85
SMU_1713c	2.11	1.08	5.71E-06	228.51	109.26
SMU_238c	2.09	1.07	0.001096	44.43	21.34
gltX	2.09	1.06	0.000144	1136.19	548.37
SMU_618	2.08	1.06	0.004251	3173.59	1544.05
SMU_1881c	2.08	1.05	0.000187	665.85	323.50
hisS	2.08	1.05	6.08E-05	957.28	464.61
gshR	2.07	1.05	0.014333	1800.68	876.09
SMU_675	2.06	1.05	0.040812	17356.41	8499.59
SMU_1710c	2.05	1.04	0.002652	51.49	25.24
SMU_2065	2.05	1.03	0.000267	455.80	225.08
SMU_1821c	2.04	1.03	0.002186	90.47	44.71
SMU_442	2.03	1.02	0.032544	215.20	107.44
SMU_2099c	2.02	1.01	0.000288	318.06	159.61
pgi	2.01	1.01	0.010202	1870.50	936.45
SMU_649	2.00	1.00	0.007144	197.23	99.33
SMU_775c	-2.00	-1.00	0.003171	406.49	819.82
fol	-2.02	-1.01	0.007195	121.30	246.28
SMU_669c	-2.02	-1.01	0.049141	36.19	73.22
SMU_191c	-2.02	-1.02	0.01863	17.13	35.07
SMU_320	-2.03	-1.02	0.000419	96.18	196.15
feoB	-2.03	-1.02	0.001002	578.22	1182.94
bacD	-2.03	-1.02	0.019281	3204.46	6591.88
miaA	-2.03	-1.02	0.010389	63.71	130.35
SMU_1802c	-2.03	-1.02	0.014388	265.19	543.80
SMU_1007	-2.04	-1.03	0.002793	148.46	305.16
gcrR	-2.05	-1.04	0.004684	465.86	964.59
SMU_393	-2.06	-1.04	0.012546	67.43	140.24
wapE	-2.06	-1.04	0.000435	278.08	578.74
SMU_207c	-2.07	-1.05	0.010389	22.83	47.71
SMU_1782	-2.07	-1.05	0.011325	60.27	125.49
murC2	-2.07	-1.05	0.001423	178.46	372.56
SMU_208c	-2.08	-1.06	0.009695	46.95	99.03
SMU_1100c	-2.08	-1.06	0.000267	126.66	266.62
serC	-2.09	-1.06	0.000432	424.11	893.84
SMU_2035	-2.13	-1.09	0.000268	186.56	401.61
SMU_2083c	-2.14	-1.10	0.001121	252.62	545.85
SMU_294	-2.14	-1.10	0.011137	15.57	33.72
glnQ	-2.15	-1.10	0.045579	254.23	549.11

SMU_636	-2.15	-1.10	0.032306	368.11	793.05
SMU_1724c	-2.15	-1.11	0.000542	24.82	53.93
SMU_758c	-2.15	-1.11	0.013062	77.14	166.56
SMU_1642c	-2.16	-1.11	0.015257	37.59	81.89
luxS	-2.17	-1.12	0.000889	101.44	220.64
SMU_1872c	-2.17	-1.12	0.000183	70.23	154.16
SMU_600c	-2.18	-1.12	0.000267	76.64	168.14
ipk	-2.20	-1.13	0.003049	55.75	123.40
kthY	-2.23	-1.16	2.18E-07	193.52	435.48
kitH	-2.23	-1.16	5.28E-05	297.80	671.32
SMU_1966c	-2.24	-1.16	0.00166	37.99	85.89
SMU_724	-2.24	-1.17	0.003171	58.88	133.30
priA	-2.25	-1.17	0.000461	516.68	1169.43
SMU_2059c	-2.25	-1.17	0.000935	54.82	124.61
SMU_1279c	-2.26	-1.18	0.000327	393.82	899.72
pstS	-2.27	-1.19	2.25E-06	368.16	846.28
SMU_71	-2.28	-1.19	0.001724	161.81	372.46
SMU_1097c	-2.30	-1.20	0.005776	53.57	123.49
SMU_1098c	-2.31	-1.21	0.006542	523.40	1219.02
SMU_197c	-2.31	-1.21	0.001525	68.56	160.73
SMU_196c	-2.31	-1.21	0.002312	31.75	74.35
SMU_1774c	-2.31	-1.21	0.007389	38.35	89.09
SMU_218	-2.38	-1.25	0.001417	11.43	27.57
glpF	-2.39	-1.25	0.000805	736.72	1775.73
SMU_217c	-2.40	-1.26	0.016071	10.09	24.50
SMU_1399	-2.40	-1.26	0.001139	31.78	76.85
SMU_198c	-2.44	-1.28	0.00097	55.21	136.39
SMU_564	-2.44	-1.29	0.00024	54.14	133.25
SMU_202c	-2.45	-1.29	0.002103	20.00	49.65
radC	-2.48	-1.31	0.00011	19.33	48.45
SMU_1249c	-2.50	-1.32	0.000544	75.27	189.50
SMU_560c	-2.57	-1.36	6.36E-07	78.71	203.86
SMU_1072c	-2.62	-1.39	0.003165	111.51	292.92
SMU_1006	-2.63	-1.39	0.000182	70.82	188.20
SMU_1046c	-2.71	-1.44	1.47E-05	198.63	545.41
SMU_1045c	-2.72	-1.44	2.3E-08	127.27	349.24
purM	-2.73	-1.45	0.021305	1382.48	3828.99
SMU_769	-2.77	-1.47	7.34E-05	13.50	37.78
SMU_1400c	-2.79	-1.48	0.000334	46.54	131.46
purN	-2.81	-1.49	0.008416	533.75	1517.29
sgaT	-2.82	-1.50	0.010337	61.02	174.35
SMU_36	-2.82	-1.50	0.003577	665.95	1903.55
SMU_1044c	-2.91	-1.54	5.37E-09	102.74	301.86
SMU_201c	-2.92	-1.55	0.000182	26.85	79.41
purH	-2.95	-1.56	0.003801	1435.71	4276.91
SMU_1071c	-2.96	-1.57	0.000155	105.27	312.28

SMU_33	-2.97	-1.57	0.004659	77.84	233.86
SMU_120	-3.00	-1.58	7.36E-05	11.73	35.61
lacG	-3.00	-1.59	0.018009	22.09	67.01
scrA	-3.04	-1.61	0.001032	321.57	985.59
purL	-3.06	-1.61	0.021047	3795.32	11681.37
dinF	-3.08	-1.62	1.54E-05	168.29	521.48
SMU_138	-3.11	-1.63	0.000577	48.40	151.84
purF	-3.14	-1.65	0.0075	1417.01	4491.88
SMU_2084c	-3.25	-1.70	1.08E-05	428.25	1403.91
mleS	-3.27	-1.71	0.000267	111.36	368.27
SMU_31	-3.46	-1.79	0.001139	191.77	669.86
SMU_753	-3.54	-1.82	0.01041	381.49	1363.66
feoA	-3.55	-1.83	8.3E-11	57.40	205.80
comYC	-3.73	-1.90	9.3E-07	13.16	49.57
ssb2	-3.75	-1.91	8.19E-09	73.64	279.30
SMU_136c	-3.75	-1.91	1.32E-08	12.68	48.18
comYB	-3.76	-1.91	5.66E-08	55.82	212.39
gidB	-3.84	-1.94	1.38E-07	43.20	167.42
SMU_299c	-3.87	-1.95	1.34E-07	16.04	62.65
SMU_1250c	-3.90	-1.96	1.2E-05	19.33	75.83
comYA	-3.98	-1.99	3.74E-10	38.23	153.64
SMU_1438c	-4.28	-2.10	7.07E-09	41.36	178.37
SMU_29	-4.94	-2.30	0.000455	180.30	890.49
SMU_145	-5.15	-2.36	7.99E-10	114.37	596.93
SMU_917c	-5.36	-2.42	4.5E-06	15.42	83.83
SMU_2057c	-5.95	-2.57	0.000268	169.21	1014.37
SMU_914c	-6.39	-2.68	1.5E-07	13.33	86.49
SMU_915c	-6.59	-2.72	6.35E-07	12.94	86.37
SMU_919c	-6.83	-2.77	6.2E-08	16.73	115.72
SMU_916c	-7.17	-2.84	1.87E-07	22.24	161.73
SMU_626	-10.91	-3.45	1.99E-18	17.10	188.76

Supplemental Table S3. Significant (FDR-adjusted p-value < 0.05) gene expression changes in LSMMG growth condition. All fold-change (FC) values represent late-exponential (LE) growth/mid-exponential (ME) growth during LSMMG growth. CPM = Counts per million (TMM-normalized).

**Gene Name**	**0 g LSMMG Fold change (LE/ME)**	**0 g LSMMG log2 Fold change (LE/ME)**	**FDR p-value**	**Average CPM (LE)**	**Average CPM (ME)**
gtfB	5.86	2.55	1.04E-19	1851.13	315.66
gbpC	4.33	2.12	5.99E-08	1286.81	296.88
citB	4.02	2.01	4.89E-06	2008.55	499.65
gatA	3.92	1.97	4.68E-11	1381.51	352.58
citZ	3.89	1.96	0.000349	1144.37	293.93
SMU_1217c	3.51	1.81	6.07E-07	225.08	64.19
adhA	3.42	1.77	0.00729	586.28	171.64
SMU_1657c	3.38	1.76	0.001721	39.61	11.73
idh	3.38	1.76	0.000725	1241.59	367.79
SMU_524	3.23	1.69	4.68E-11	289.82	89.61
SMU_706c	3.18	1.67	1.98E-06	43.38	13.62
SMU_673	3.12	1.64	3.92E-05	473.25	151.76
nrgA	3.11	1.63	0.003185	146.54	47.18
sloA	3.02	1.59	0.012951	248.55	82.34
SMU_929c	2.95	1.56	0.000417	471.34	159.55
pyrAB	2.93	1.55	2.18E-05	2855.04	973.20
SMU_525	2.91	1.54	3.99E-09	351.78	120.97
SMU_1821c	2.82	1.50	5.61E-06	204.59	72.54
opuAb	2.80	1.48	2.44E-05	2937.81	1049.90
pyrDB	2.78	1.48	5.71E-05	347.92	125.15
naoX	2.76	1.46	0.000633	1040.60	377.19
argC	2.73	1.45	0.003591	46.61	17.04
pyrA	2.72	1.44	2.44E-05	712.33	262.25
SMU_2109	2.71	1.44	0.000176	162.49	59.90
topA	2.67	1.42	1.94E-06	1206.79	451.46
glnP	2.63	1.40	1.54E-07	632.86	240.46
pyrK	2.58	1.37	0.001076	182.15	70.65
pyrB	2.58	1.37	0.000152	526.27	204.19
SMU_144c	2.55	1.35	0.000202	200.43	78.75
opuAa	2.54	1.35	0.000423	1740.68	684.26
SMU_2104a	2.51	1.33	2.44E-05	304.68	121.58
SMU_541	2.50	1.32	0.002406	38.83	15.55
SMU_1571	2.41	1.27	0.007859	148.49	61.55
SMU_105	2.41	1.27	0.042446	1322.97	549.94
SMU_613	2.40	1.26	0.00287	114.83	47.87
SMU_1287	2.37	1.25	0.041256	28.91	12.18
SMU_1220c	2.37	1.24	0.00146	242.00	102.33
fruB	2.34	1.22	0.014839	67.97	29.12

dexB	2.33	1.22	0.011796	201.26	86.42
SMU_1820c	2.30	1.20	0.000179	1845.20	801.71
SMU_954	2.29	1.20	0.005448	49.51	21.60
gatB	2.29	1.19	0.001738	1920.73	839.84
SMU_1521	2.28	1.19	3.92E-05	767.69	336.64
gid	2.28	1.19	0.000234	848.80	372.82
argJ	2.27	1.18	0.003185	92.25	40.58
SMU_774	2.23	1.16	0.00323	206.43	92.44
SMU_704c	2.23	1.16	0.000252	104.79	46.98
SMU_857	2.23	1.16	0.001267	608.58	273.19
SMU_1219c	2.20	1.14	0.000719	67.36	30.63
SMU_1351	2.20	1.14	0.000725	33.52	15.25
malG	2.16	1.11	0.009886	78.99	36.49
pyrE	2.15	1.11	0.000481	381.97	177.61
SMU_238c	2.15	1.10	0.001076	51.29	23.87
SMU_1710c	2.12	1.09	0.00225	92.93	43.78
gidA	2.12	1.08	0.000269	593.82	280.45
SMU_301	2.11	1.08	0.002263	175.05	83.15
nylA	2.11	1.07	0.000158	338.55	160.82
mutY	2.10	1.07	0.000831	183.88	87.73
SMU_1637c	2.09	1.06	0.015009	44.36	21.29
tgt	2.08	1.05	9.6E-06	307.27	147.98
SMU_1405c	2.07	1.05	0.001076	556.05	268.62
SMU_382c	2.06	1.04	0.02395	20.98	10.21
SMU_1064c	2.06	1.04	0.042548	74.93	36.39
SMU_1520	2.06	1.04	0.012712	777.07	377.80
SMU_16	2.05	1.03	0.000655	409.72	200.13
SMU_1116c	2.03	1.02	0.006032	761.85	374.55
SMU_1403c	2.03	1.02	0.010149	49.29	24.24
SMU_1402c	2.03	1.02	0.027128	42.68	21.04
pyrF	2.02	1.02	0.001049	412.10	203.56
psaR	2.02	1.02	0.013979	71.48	35.33
SMU_1284c	2.02	1.01	0.011904	78.89	39.06
SMU_169	2.02	1.01	0.040481	2476.46	1228.82
SMU_1397c	2.01	1.01	0.02177	60.16	29.88
trcF	2.01	1.01	9.01E-05	557.66	277.51
argB	1.99	0.99	0.020461	55.79	28.09
pth	1.98	0.99	0.002017	77.51	39.17
SMU_1327c	1.98	0.99	0.003456	105.13	53.14
SMU_521	1.98	0.98	0.011308	206.63	104.50
SMU_61	1.97	0.98	0.030445	196.33	99.65
SMU_1636c	1.96	0.97	0.00151	272.09	138.79
SMU_955	1.96	0.97	0.013979	46.01	23.50
SMU_170	1.95	0.97	0.020461	2454.72	1257.52
SMU_1041	-1.96	-0.97	0.002568	110.98	217.42
SMU_136c	-1.98	-0.98	0.010391	27.87	55.09

bcc	-1.98	-0.99	0.00364	55.04	109.15
gbpA	-1.99	-0.99	0.018606	856.72	1703.27
SMU_2035	-2.00	-1.00	0.001688	239.44	478.35
SMU_995	-2.00	-1.00	0.00837	268.80	538.34
gbpD	-2.01	-1.01	0.000505	161.47	325.32
SMU_993	-2.05	-1.04	0.000633	230.46	472.79
SMU_807	-2.06	-1.04	0.018501	81.34	167.28
SMU_992	-2.11	-1.07	0.000113	166.70	351.17
SMU_644	-2.11	-1.07	0.016567	14.16	29.87
sacB	-2.11	-1.08	0.004462	748.97	1580.57
feoB	-2.11	-1.08	0.000822	651.44	1374.72
prs	-2.11	-1.08	0.000223	422.09	891.00
serC	-2.12	-1.08	0.000603	606.58	1285.02
SMU_196c	-2.19	-1.13	0.007923	48.63	106.47
SMU_1896c	-2.20	-1.14	0.018789	22.71	49.91
SMU_195c	-2.21	-1.15	0.04275	10.87	24.08
SMU_138	-2.26	-1.18	0.030445	43.61	98.50
SMU_60	-2.28	-1.19	0.001064	81.69	186.34
feoA	-2.41	-1.27	2.44E-05	112.67	271.79
fthS	-2.46	-1.30	0.0036	1292.76	3178.15
SMU_299c	-2.50	-1.32	0.001116	17.52	43.73
SMU_207c	-2.53	-1.34	0.000992	35.34	89.39
SMU_198c	-2.58	-1.36	0.000714	116.60	300.25
mleS	-2.58	-1.37	0.008317	81.88	211.44
SMU_197c	-2.65	-1.40	0.000317	124.97	330.66
SMU_55	-2.70	-1.43	0.003185	86.87	234.17
SMU_58	-2.70	-1.43	5.71E-05	269.03	725.45
glgA	-2.75	-1.46	0.034279	302.74	832.76
SMU_435	-2.78	-1.47	0.00837	224.52	623.83
SMU_52	-2.89	-1.53	4.88E-05	226.10	653.36
SMU_53	-2.90	-1.53	0.000175	84.38	244.37
SMU_208c	-2.94	-1.55	0.000102	63.69	187.14
sgaT	-2.95	-1.56	0.011308	106.76	314.63
SMU_1895c	-3.04	-1.60	6.73E-05	10.93	33.30
SMU_209c	-3.05	-1.61	0.000202	16.30	49.70
purB	-3.09	-1.63	6E-05	718.75	2224.02
SMU_54	-3.12	-1.64	5.71E-05	270.60	844.57
purD	-3.18	-1.67	0.000421	687.56	2185.83
SMU_49	-3.28	-1.72	2.44E-05	404.02	1326.46
purK	-3.30	-1.72	0.000579	599.53	1977.38
SMU_202c	-3.32	-1.73	3.71E-05	21.75	72.09
SMU_199c	-3.34	-1.74	2.18E-05	16.67	55.69
SMU_201c	-3.41	-1.77	2.44E-05	43.79	149.36
purE	-3.49	-1.80	5.61E-06	145.34	506.97
SMU_917c	-3.50	-1.81	0.001575	37.73	132.23
SMU_919c	-3.64	-1.86	0.000901	55.12	200.41

SMU_1072c	-3.71	-1.89	5.71E-05	44.42	164.53
glgB	-3.72	-1.89	0.021824	801.49	2978.41
SMU_916c	-3.75	-1.91	0.001493	72.91	273.39
SMU_915c	-3.86	-1.95	0.001035	50.33	194.33
SMU_914c	-3.90	-1.96	0.000366	46.42	180.94
glgD	-3.92	-1.97	0.002874	186.92	732.63
SMU_29	-4.03	-2.01	0.004571	138.72	559.26
ssb2	-4.26	-2.09	3.8E-10	43.65	185.91
SMU_2057c	-4.29	-2.10	0.006694	475.53	2041.78
SMU_36	-4.38	-2.13	3.18E-05	409.39	1791.38
SMU_33	-4.47	-2.16	9.45E-05	51.87	231.96
comYD	-4.47	-2.16	2.13E-06	13.48	60.35
purH	-4.50	-2.17	5.65E-05	1000.02	4502.10
purM	-4.51	-2.17	0.000433	648.39	2925.29
purN	-4.53	-2.18	9.45E-05	332.41	1504.20
comYA	-4.55	-2.18	6.92E-12	29.35	133.50
SMU_31	-4.62	-2.21	8.25E-05	174.65	806.50
comYB	-4.62	-2.21	3.84E-10	36.14	167.02
purL	-4.65	-2.22	0.00143	2090.35	9723.56
glgC	-4.78	-2.26	0.00112	296.81	1419.84
purF	-5.06	-2.34	0.000132	878.93	4443.43
bacT	-5.42	-2.44	1.09E-09	492.43	2667.43
SMU_1345c	-6.11	-2.61	1.6E-09	1189.41	7263.37
comEA	-6.40	-2.68	1.44E-17	11.80	75.62
SMU_1344c	-6.50	-2.70	4.68E-11	453.18	2947.12
SMU_1343c	-7.06	-2.82	1.6E-10	1073.16	7577.98
SMU_626	-7.27	-2.86	1.54E-12	27.47	199.59
bacA1	-8.24	-3.04	4.52E-11	1961.91	16162.91
SMU_1341c	-9.45	-3.24	3.09E-15	988.15	9337.51
bacD	-9.51	-3.25	2.12E-17	959.38	9124.61
bacA2	-10.18	-3.35	3.17E-16	1307.29	13308.00

Supplemental Table S4. Significant (FDR-adjusted p-value < 0.05) gene expression changes in NG growth condition. All fold-change (FC) values represent late-exponential (LE) growth/mid-exponential (ME) growth during NG growth. CPM = Counts per million (TMM-normalized).

Gene Name	NG Fold change (LE/ME)	NG log2 Fold change (LE/ME)	FDR p-value	Average CPM (LE)	Average CPM (ME)
SMU_105	7.79	2.96	1.48E-08	3192.27	411.78
SMU_574c	7.24	2.86	0.002424	653.65	90.47
SMU_575c	6.58	2.72	0.001218	456.73	69.72
SMU_104	6.30	2.65	0.000102	7518.80	1198.89
pdhC	6.15	2.62	0.000254	272.94	44.53
dexB	5.71	2.51	2.48E-09	458.16	80.54
SMU_1956c	5.54	2.47	0.00019	901.98	163.64
msmK	5.47	2.45	7.36E-06	287.03	52.71
SMU_1957	4.94	2.30	0.001821	2971.23	604.24
malG	4.78	2.26	2.38E-09	149.66	31.43
SMU_103	4.76	2.25	0.002551	1146.80	242.20
gtfA	4.60	2.20	2.13E-05	321.83	70.26
SMU_1571	4.60	2.20	2.83E-07	267.82	58.49
SMU_1958c	4.45	2.16	0.007959	2948.87	665.53
msmG	4.34	2.12	0.00015	157.96	36.55
pdhB	4.25	2.09	0.009847	223.67	52.74
adhA	4.08	2.03	0.001701	623.35	153.15
SMU_1961c	3.91	1.97	0.033048	496.01	127.40
naoX	3.78	1.92	2.75E-06	1137.16	302.10
SMU_102	3.74	1.90	0.02221	3091.30	829.18
ptcC	3.73	1.90	0.007808	74.81	20.15
SMU_1821c	3.71	1.89	2.38E-09	208.12	56.34
gbpC	3.66	1.87	2.75E-06	990.62	271.49
gatA	3.63	1.86	8.71E-10	1363.08	376.88
fruB	3.51	1.81	9.81E-05	85.74	24.51
grpE	3.50	1.81	0.015756	129.82	37.26
SMU_1402c	3.48	1.80	1.06E-05	50.90	14.69
malF	3.36	1.75	0.000448	270.13	80.64
SMU_1862	3.33	1.74	0.043862	45.23	13.63
fruA	3.30	1.72	0.008597	1797.54	548.42
gtfB	3.11	1.64	3.07E-08	1156.06	374.02
hrcA	3.10	1.63	0.013697	297.89	96.49
msmE	3.07	1.62	0.016724	225.56	73.83
SMU_1116c	3.05	1.61	2.75E-06	903.66	297.81
SMU_1906c	2.99	1.58	0.020384	131.77	44.09
msmF	2.99	1.58	0.005309	171.55	57.50
dnaK	2.91	1.54	0.019353	1350.52	465.97
SMU_1217c	2.89	1.53	4.51E-05	161.91	56.29
SMU_1913c	2.78	1.48	0.001516	631.97	227.84
SMU_144c	2.78	1.47	4.05E-05	205.63	74.30

pttB	2.77	1.47	0.001498	124.85	45.18
SMU_1048	2.73	1.45	0.004548	41.90	15.43
glnP	2.71	1.44	6.08E-08	451.92	167.69
opuAb	2.64	1.40	8.24E-05	2751.34	1046.43
pyrAB	2.64	1.40	0.000169	2591.03	985.55
SMU_1405c	2.59	1.37	8.17E-06	651.68	253.20
SMU_1403c	2.59	1.37	0.00028	58.84	22.86
SMU_1404c	2.55	1.35	0.000568	160.42	63.07
treA	2.52	1.33	0.000811	91.60	36.57
adhB	2.51	1.33	0.04032	1036.78	415.07
SMU_768c	2.48	1.31	0.003657	47.19	19.13
dnaJ	2.45	1.29	0.018226	458.65	187.80
SMU_525	2.41	1.27	3.32E-06	239.05	99.79
lytR	2.39	1.25	0.017866	186.88	78.69
groES	2.38	1.25	0.0324	144.60	60.89
SMU_1820c	2.37	1.25	0.000103	1960.87	829.33
galR	2.37	1.25	0.002551	54.85	23.22
pyrA	2.33	1.22	0.000568	660.11	284.65
SMU_524	2.31	1.21	9.3E-06	182.80	79.44
SMU_100	2.30	1.20	0.010843	1472.35	642.49
purR	2.29	1.20	0.002222	581.50	254.88
SMU_1909c	2.28	1.19	0.042211	660.38	290.12
SMU_1710c	2.27	1.18	0.000873	82.00	36.25
SMU_405c	2.27	1.18	0.013302	115.52	51.14
SMU_1064c	2.26	1.18	0.019353	76.87	34.20
adhC	2.24	1.17	0.028892	1313.32	587.41
gatB	2.24	1.16	0.00293	2011.65	902.68
treR	2.20	1.14	0.009472	24.47	11.16
SMU_874	2.18	1.13	0.000567	66.53	30.60
lytS	2.18	1.12	0.044556	510.23	235.22
SMU_242c	2.16	1.11	0.010479	422.90	196.77
SMU_1713c	2.15	1.11	7.19E-06	304.49	142.00
SMU_1511c	2.15	1.10	0.000911	304.97	142.47
galK	2.15	1.10	0.03368	85.65	40.07
coaA	2.15	1.10	0.001232	102.00	47.75
sloC	2.14	1.10	0.033427	65.39	30.62
SMU_1397c	2.14	1.10	0.012258	55.72	26.11
pyrB	2.14	1.10	0.00403	474.28	222.82
SMU_1220c	2.12	1.09	0.008723	194.23	91.91
SMU_161	2.12	1.09	0.000568	227.36	107.61
mutY	2.12	1.09	0.000809	165.45	78.33
SMU_125	2.10	1.07	0.003559	286.85	137.41
syt1	2.09	1.06	0.001764	1283.08	616.91
opuAa	2.05	1.04	0.012333	1592.42	779.59
SMU_238c	2.03	1.02	0.003559	42.79	21.12
SMU_1714c	2.02	1.01	0.00028	280.03	139.52

SMU_2027	1.98	0.98	0.035063	148.00	75.22
SMU_503c	1.97	0.98	0.020729	176.41	90.18
SMU_162c	1.96	0.97	0.016343	145.87	74.82
SMU_1637c	1.96	0.97	0.0324	35.55	18.23
galT	1.95	0.97	0.042526	183.36	94.19
SMU_754	-1.95	-0.96	0.015734	1919.01	3757.89
pstS	-1.96	-0.97	0.000375	397.11	781.11
SMU_1507c	-1.97	-0.98	0.02689	17.11	33.86
SMU_294	-1.98	-0.99	0.038915	15.60	31.04
SMU_2079c	-1.99	-0.99	0.02491	252.58	503.93
gcrR	-2.00	-1.00	0.013329	478.34	959.71
SMU_145	-2.01	-1.00	0.0324	295.89	596.74
pstC	-2.01	-1.01	0.003509	412.46	831.80
SMU_1098c	-2.01	-1.01	0.044011	415.13	837.14
SMU_481	-2.02	-1.02	0.002511	290.84	590.76
metK	-2.03	-1.02	5.94E-05	511.95	1045.41
glpF	-2.03	-1.02	0.015295	929.08	1897.00
purB	-2.07	-1.05	0.020187	1205.68	2508.65
gbpD	-2.08	-1.05	0.000289	147.25	307.32
SMU_1895c	-2.08	-1.06	0.017806	12.19	25.53
pstC1	-2.10	-1.07	0.001355	436.05	919.60
SMU_1545c	-2.11	-1.08	0.009472	91.41	193.63
SMU_1046c	-2.11	-1.08	0.003657	217.37	460.63
SMU_1896c	-2.13	-1.09	0.026865	22.73	48.62
SMU_71	-2.13	-1.09	0.00898	107.43	230.09
SMU_1876	-2.15	-1.10	0.010843	269.19	580.57
SMU_1314	-2.16	-1.11	0.000911	358.64	777.78
kguA	-2.21	-1.14	0.036681	852.97	1888.58
SMU_993	-2.21	-1.14	0.000146	213.47	474.04
SMU_36	-2.25	-1.17	0.044556	683.50	1546.92
SMU_1642c	-2.26	-1.18	0.016576	34.12	77.50
SMU_836	-2.26	-1.18	0.001199	57.29	130.35
SMU_277	-2.27	-1.18	0.009512	79.01	179.77
kitH	-2.29	-1.19	6.63E-05	228.95	526.07
SMU_992	-2.33	-1.22	8.06E-06	142.23	332.27
SMU_198c	-2.33	-1.22	0.003657	95.91	224.48
SMU_33	-2.39	-1.26	0.045054	84.24	202.07
SMU_1502c	-2.39	-1.26	0.000332	35.20	84.50
purN	-2.42	-1.28	0.044011	540.52	1314.31
purH	-2.45	-1.29	0.033427	1577.53	3883.42
SMU_207c	-2.46	-1.30	0.001821	28.53	70.51
SMU_208c	-2.51	-1.33	0.001523	57.92	145.98
SMU_209c	-2.51	-1.33	0.003698	13.62	34.38
sgaT	-2.56	-1.35	0.035183	133.63	343.63
SMU_197c	-2.61	-1.39	0.000448	99.44	261.37
SMU_196c	-2.75	-1.46	0.000331	33.53	92.78

SMU_299c	-2.81	-1.49	0.000206	15.95	45.06
purM	-2.85	-1.51	0.02689	1024.96	2926.60
SMU_462	-2.88	-1.53	0.000911	54.58	158.01
SMU_915c	-2.96	-1.57	0.014292	49.38	146.94
SMU_201c	-3.06	-1.61	0.000168	39.69	121.99
SMU_199c	-3.12	-1.64	7.35E-05	13.94	43.77
SMU_914c	-3.16	-1.66	0.004025	45.36	144.12
SMU_917c	-3.45	-1.79	0.002277	35.31	122.31
SMU_916c	-3.58	-1.84	0.002854	69.87	251.24
bacT	-3.67	-1.88	8.06E-06	639.16	2358.54
SMU_919c	-3.80	-1.93	0.000647	48.80	186.36
mreD	-3.86	-1.95	0.000207	53.38	206.72
SMU_202c	-4.24	-2.09	2.59E-07	17.26	73.67
SMU_753	-4.28	-2.10	0.004884	233.74	1003.31
ssb2	-4.59	-2.20	4.03E-11	50.36	232.27
SMU_1344c	-4.60	-2.20	2.43E-07	520.88	2406.09
comYD	-4.62	-2.21	1.15E-06	10.38	48.36
SMU_1345c	-4.69	-2.23	5.63E-07	1449.55	6829.47
SMU_1343c	-5.00	-2.32	3.47E-07	1254.89	6300.11
comYA	-5.45	-2.45	9.99E-15	23.66	129.72
bacA1	-5.70	-2.51	1.36E-07	2216.19	12681.01
comYB	-6.12	-2.61	7.54E-14	28.83	177.25
bacD	-6.35	-2.67	1.36E-11	1035.56	6601.62
SMU_1341c	-6.53	-2.71	1.29E-10	1090.34	7155.81
SMU_2057c	-6.59	-2.72	0.000238	273.39	1806.96
bacA2	-6.97	-2.80	2.66E-11	1466.52	10258.01
SMU_626	-10.58	-3.40	1.36E-17	18.39	195.83
